# Taming Prolonged Ionic Drift–Diffusion Dynamics for Brain‐Inspired Computation

**DOI:** 10.1002/adma.202407326

**Published:** 2024-11-27

**Authors:** Hisashi Inoue, Hiroto Tamura, Ai Kitoh, Xiangyu Chen, Zolboo Byambadorj, Takeaki Yajima, Yasushi Hotta, Tetsuya Iizuka, Gouhei Tanaka, Isao H. Inoue

**Affiliations:** ^1^ National Institute of Advanced Industrial Science and Technology (AIST) Tsukuba 305‐8565 Japan; ^2^ Graduate Schools for Law and Politics The University of Tokyo Tokyo 113‐0033 Japan; ^3^ International Research Center for Neurointelligence (IRCN) The University of Tokyo Tokyo 113‐0033 Japan; ^4^ Systems Design Laboratory School of Engineering, The University of Tokyo Tokyo 113‐0032 Japan; ^5^ Graduate School of Information Science and Electrical Engineering Kyushu University Fukuoka 819‐0395 Japan; ^6^ Department of Engineering University of Hyogo Hyogo 671‐2280 Japan; ^7^ Department of Computer Science Nagoya Institute of Technology Nagoya 466‐8555 Japan

**Keywords:** drift–diffusion, leaky integration, neural network, oxygen vacancy, reservoir computing

## Abstract

Recent advances in neural network‐based computing have enabled human‐like information processing in areas such as image classification and voice recognition. However, many neural networks run on conventional computers that operate at GHz clock frequency and consume considerable power compared to biological neural networks, such as human brains, which work with a much slower spiking rate. Although many electronic devices aiming to emulate the energy efficiency of biological neural networks have been explored, achieving long timescales while maintaining scalability remains an important challenge. In this study, a field‐effect transistor based on the oxide semiconductor strontium titanate (SrTiO_3_) achieves leaky integration on a long timescale by leveraging the drift–diffusion of oxygen vacancies in this material. Experimental analysis and finite‐element model simulations reveal the mechanism behind the leaky integration of the SrTiO_3_ transistor. With a timescale in the order of one second, which is close to that of biological neuron activity, this transistor is a promising component for biomimicking neuromorphic computing.

## Graphical abstract

To mimic the energy efficiency of biological neural networks, an artificial neural device that works on long timescales with minimal energy consumption is needed. By leveraging the drift–diffusion dynamics of oxygen vacancies, a field‐effect transistor made of an oxide semiconductor achieves a timescale of the order of one second, comparable to that of biological neurons, with negligible power loss.

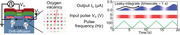

## 1 Introduction

In the realm of neural computation, achieving efficient information processing analogous to that realized by biological systems remains a paramount challenge. Particularly in applications such as speech recognition and motion detection with temporal dynamics of the order of milliseconds to seconds, biological neural networks outperform modern computers, even though the processing timescale of the former is much longer than that of the latter.^[^ ^1^, ^2^, ^3^ ^]^ Despite advances in deep‐learning frameworks, which typically run on von Neumann computer architectures, the energy costs remain prohibitively high for real‐time performance on portable edge devices, necessitating the exploration of novel architectures and devices to realize energy‐efficient practical neural computing.

**Figure 1:**
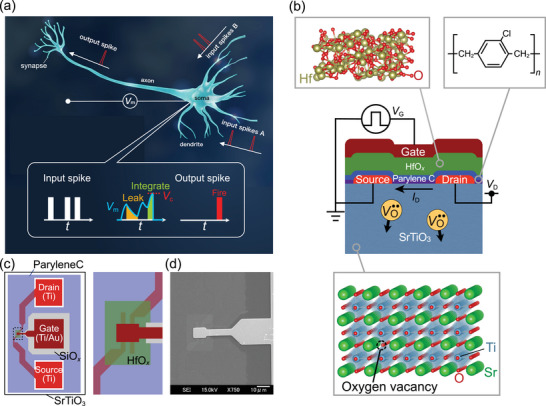
Biological neuron operation and SrTiO_3_‐based leaky‐integration FET. a) Schematic illustration of a neuron cell. Inset: schematic diagram of leaky integration. b) Schematic illustration (side view) of a SrTiO_3_‐based leaky‐integration FET. Application of gate voltage *V*_G_ controls the drift–diffusion dynamics of oxygen vacancies $V_{O}^{..}$, which modulates the induced drain current *I*_D_ within the SrTiO_3_ crystal (bottom panel). The gate dielectric of the transistor consists of Parylene C and amorphous HfO_*x*_ layers (top panels). c) Schematic diagram of the top view of the SrTiO_3_‐based FET. The right panel is a magnified view of the dashed rectangle in the left panel. d) Scanning electron microscope image of the FET.

Neuromorphic computing, inspired by the efficiency of biological neural networks exchanging spikes between neurons (**Figure** **1**a), seeks to replicate this capability using interconnected artificial neurons.^[^ ^4^ ^]^ Biological neural networks operate on timescales ranging from ≈1 ms up to ≈10 s.^[^ ^5^ ^]^ Focusing on the slow dynamics of ≈0.1 to 1 s, it is understood that neural networks adjust their dynamics to align with the timescales of incoming signals, enabling efficient real‐time information processing with low energy consumption. The energy efficiency of neural networks comes from the fact that most energy is consumed only when there is a spike, unlike conventional complementary metal–oxide–semiconductor (CMOS) integrated circuits, which consume a considerable amount of energy even when no signal is being processed. However, many studies on artificial neural networks currently focus on networks and devices that work at timescales much faster than that of biological neural networks.^[^ ^6^, ^7^, ^8^ ^]^ When the timescale of a neural network is much faster than the incoming signals, the signals must first be stored in an external memory and then processed serially by taking them out of the memory. In this case, additional energy is needed for memory access, as in conventional computers, resulting in increased energy consumption. However, by designing artificial neurons to operate on timescales comparable to those of biological neural networks (i.e., up to 10 s), the need for frequent memory access is alleviated. Instead of continuously fetching and updating information from a memory, a neural network with a suitable timescale can retain and process sensory inputs over extended periods with minimal energy consumption.

The leaky‐integration behavior of neurons is a critical characteristic that determines the timescales of neural networks. An input pulse at the dendrites increases or decreases the electrical potential of the soma, which is called the membrane potential (*V*_m_). The change of *V*_m_ accumulates, or integrates, when subsequent pulses arrive at the soma. Conversely, *V*_m_ gradually decays — that is, leaks — to its resting value without subsequent pulses. When *V*_m_ reaches a threshold value of *V*_c_, a pulse is generated from the axon and the information is transferred to connecting post‐synaptic neurons. Various electronic devices and technologies, such as CMOS circuits,^[^ ^9^, ^10^, ^11^, ^12^, ^13^, ^14^, ^15^, ^16^ ^]^ resistive random‐access memories,^[^ ^17^, ^18^, ^19^, ^20^, ^21^, ^22^, ^23^, ^24^, ^25^ ^]^ ferroelectric devices,^[^ ^26^, ^27^, ^28^, ^29^, ^30^, ^31^ ^]^ spintronic devices,^[^ ^32^, ^33^ ^]^ and phase change memories,^[^ ^34^, ^35^, ^36^, ^37^ ^]^ have been explored to mimic this behavior. Furthermore, two‐terminal memristive leaky‐integration devices with high reliability and uniform characteristics demonstrating various functionalities have been reported recently.^[^ ^38^ ^]^ However, it is still challenging to achieve long timescales while maintaining energy efficiency and scalability.^[^ ^39^ ^]^ Taking CMOS as an example, a timescale of 1 s corresponds to a capacitance of 1 µF, which takes up considerable space on a die. Furthermore, the energy to drive such devices is substantial because the amount of energy generally increases as the timescale lengthens. Although many devices focus on nonvolatile operation,^[^ ^40^, ^41^, ^42^, ^43^ ^]^ this nonvolatility makes the implementation of leaky integration difficult because an external mechanism for leakage is needed.

The drift–diffusion of ions is advantageous for generating long timescales because of the slower motion of ions than that of electrons. Drift–diffusion of ions is a general phenomenon used in various applications, and these devices typically work on long timescales. Charged ions can be controlled by applied electric fields, but their material‐specific diffusion coefficient generally limits their migration speed, and their motion is dynamic and reversible. This means that achieving a long timescale is possible without compromising energy efficiency, device size, or volatility. We report the development of an artificial neuron device with leaky integration, as shown in Figure 1b,c, based on the drift–diffusion of oxygen vacancies ($V_{O}^{..}$) in SrTiO_3_, a well‐known oxide semiconductor.^[^ ^44^, ^45^ ^]^ $V_{O}^{..}$ are defects where oxygen atoms are missing in the crystal lattice. $V_{O}^{..}$ exert a profound influence on the electrical and ionic transport properties of materials. Electrostatically controlling the drift–diffusion dynamics of $V_{O}^{..}$ in SrTiO_3_ is attractive because of its large relative permittivity of 300 and the relatively small electron densities required to induce electrical conductivity. By harnessing the drift–diffusion dynamics of $V_{O}^{..}$, we demonstrate the feasibility of achieving leaky integration over timescales relevant to real‐world information processing tasks, such as speech and motion analysis. Furthermore, we present a comprehensive investigation of the operation principles of our proposed device, combining experimental results with finite element simulations to elucidate the underlying mechanisms.

## 2 Results and Discussion

### 2.1 Handwriting Anomaly Detection and Effect of Timescales

**Figure 2:**
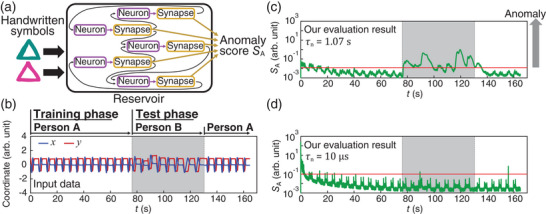
Effect of neuron timescale on brain‐inspired computing. a) Schematic illustration of reservoir computing‐based anomaly detection. b) Handwriting trajectory data used for evaluation. c,d) Anomaly scores calculated from the reservoir activity when handwriting trajectories were input. The timescales of the neural elements are (c) *τ*_n_ = 1.07 s and (d) *τ*_n_ = 10 µs.

The effect of timescale on the performance of a neural network is exemplified in the task of handwriting anomaly detection shown in **Figure** **2**a. The task was to distinguish symbols (e.g., triangles) drawn by different people. The neural network received time‐varying trajectories of a pen and determined which person drew each triangle^[^ ^46^ ^]^ (see the Experimental Section and Section S4, Supporting Information). The time‐dependent trajectory (i.e., *x*, *y* coordinates shown in Figure 2b) of a pen was processed in the framework of reservoir computing depicted schematically in Figure 2a.^[^ ^47^, ^48^, ^49^ ^]^ The randomly interconnected neural network, called a reservoir, received temporal inputs and encoded shape characteristics of the trajectories into the nonlinear dynamics of constituting neurons, which in turn determined the habits of each person drawing the symbols. Figure 2c shows the anomaly score *S*_A_ (i.e., Mahalanobis distance of the reservoir state; see Section S4, Supporting Information) that quantifies the difference between the trajectories of the training and testing phases. Here, the neural network was trained only with person A's trajectory and tested against the different trajectories of person A and person B. When only person B's trajectory was provided, *S*_A_ increased by orders of magnitude. Therefore, handwriting anomaly detection was successful when the timescale of neurons *τ*_n_ was 1.07 s. Surprisingly, this contrasts with the case when fast neurons (*τ*_n_ = 10 µs, e.g., CMOS neurons, see Section S3, Supporting Information) were used, where handwriting anomaly detection was unsuccessful, as shown in Figure 2d (see Section S10, Supporting Information for a discussion of the effect of neuron timescale on information processing performance). The results clearly indicate the timescale of the neuron is an important factor that determines the performance of real‐time neuromorphic computing for signals that have long timescales (e.g., voice and motion).

### 2.2 Long‐Timescale Leaky‐Integration FET

**Figure 3:**
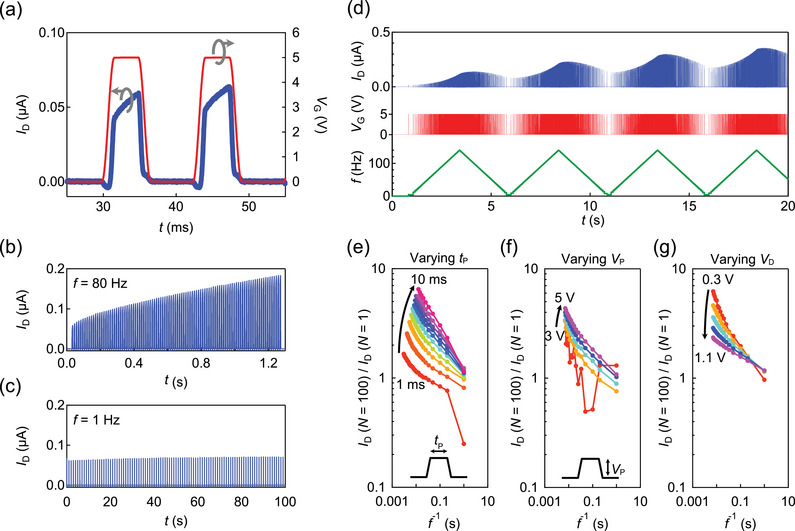
Leaky integration in the SrTiO_3_ FET. a) The drain current *I*_D_ (blue) started to flow when gate pulse *V*_G_ (red) was applied to SrTiO_3_ FET. *I*_D_ gradually increased during *V*_G_ application, demonstrating leaky integration. b,c) Leaky integration of trains of pulses using the SrTiO_3_ FET. The amplitude of *I*_D_ increased after every pulse at (b) *f* = 80 Hz, but it did not increase at (c) *f* = 1 Hz. d) Frequency modulation of the drain current. The amplitude of *I*_D_ (blue) increased or decreased in response to the frequency change (green) of the gate pulses (red). e,f,g) Leaky‐integration rate *I*_D_(*N* = 100)/*I*_D_(*N* = 1) as a function of the inverse frequency of gate pulses when the (e) pulse width *t*_p_, (f) pulse voltage *V*_p_, and (g) source–drain voltage *V*_D_ were varied.

The SrTiO_3_ field‐effect transistor (FET) was fabricated on a single‐crystal SrTiO_3_ substrate using standard photolithography (Figure 1b–d).^[^ ^46^, ^50^, ^51^ ^]^ Details of device fabrication are provided in the Experimental Section. **Figure** **3**a depicts the time evolution of the source–drain current (*I*_D_) of the transistor under 5‐V gate pulses with a fixed source–drain voltage (*V*_D_) of 0.5 V. Notably, *I*_D_ gradually increased after the FET was turned on, despite the fixed 5‐V gate voltage (*V*_G_) during the pulses. The transient drop (rise) of *I*_D_ on the rising (falling) edge of the *V*_G_ pulse originated from the displacement currents caused by the charging (discharging) of the drain‐to‐gate and drain‐to‐source capacitance.

To examine the transistor's response across different timescales, Figure 3b,c display the time evolution of *I*_D_ during a series of gate pulses with varying pulse frequencies (*f*). At *f* = 80 Hz, the overall amplitude of *I*_D_ gradually increased, whereas at *f* = 1 Hz, the amplitude remained relatively constant after repeated gate pulses. This frequency‐dependent behavior is further illustrated in Figure 3d, which shows that frequency varied continuously over time. Here, the amplitude of *I*_D_ followed the frequency modulation: higher frequencies led to quicker amplitude increases, whereas lower frequencies led to faster amplitude decay.

To further quantify the rate of *I*_D_ increase during gate pulses, Figure 3e,f,g illustrate the ratio of *I*_D_ during the 100th pulse to *I*_D_ during the first pulse across the parameter space defined by frequency and pulse width (*t*_p_), pulse voltage (*V*_p_), and *V*_D_, respectively. Notably, for a given *t*_p_, the ratio increased monotonically with frequency. Similarly, the ratio increased monotonically with *t*_p_ at a fixed frequency. Thus, the on–drain current is influenced by not only the duration of the gate pulse but also the interval between gate pulses.

These observations provide two important insights. First, the SrTiO_3_ FET possesses an internal parameter that governs *I*_D_. Second, this internal parameter integrates upon application of gate voltage and leaks upon its release, thereby exemplifying a leaky‐integration function. Notably, the leaky‐integration rate remains nearly constant with integration timescales consistently between 10 ms and 1 s, even when varying *V*_p_ and *V*_D_. The insensitivity of the leaky‐integration rate to these parameters arises from the mechanism of leaky integration, as discussed below.

### 2.3 Long‐Timescale Dynamics of Oxygen Vacancies in the Transistor

**Figure 4:**
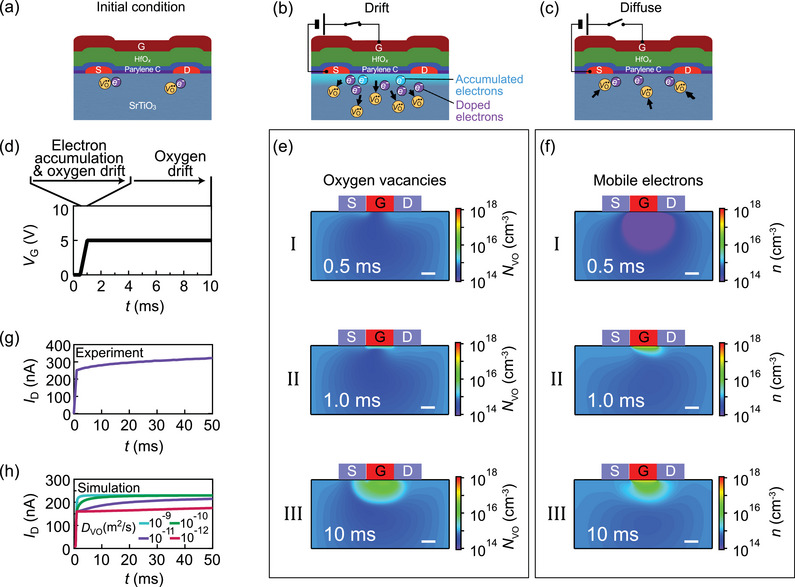
Oxygen drift–diffusion in the leaky‐integration SrTiO_3_ FET. a,b,c) Schematic illustration of the drift–diffusion of $V_{O}^{..}$. The gate, source, and drain electrodes are labeled G, S, and D, respectively. d) Schematic diagram of the *V*_G_ ramp used in the analysis. e) Snapshots of $V_{O}^{..}$ distribution at different time steps during drift–diffusion. f) Snapshots of the mobile electron distribution at different time steps during drift–diffusion. The scale bars in e and f are 1 µm. g) Time evolution of drain currents measured for the SrTiO_3_ FET. h) Time evolution of drain currents calculated for different $V_{O}^{..}$ diffusion coefficients in SrTiO_3_. Drain current continued to increase even after *V*_G_ stabilized at *t* = 1.0 ms.

The long timescale of the FET in the order of one second implies the presence of prolonged dynamics beyond electron dynamics within the system. As we explain below, the leaky‐integration behavior originates from the drift–diffusion of $V_{O}^{..}$ across the SrTiO_3_ channel. Upon fabrication of the FET, SrTiO_3_ accommodates a nominal concentration of $V_{O}^{..}$ on the order of ≈10^15^ cm^−3^ dispersed throughout its structure, primarily originating from crystal defects and impurities (**Figure** **4**a).^[^ ^52^ ^]^ These $V_{O}^{..}$, which can be considered positively charged ions, migrate in response to applied gate voltage and behave as donors to the semiconducting SrTiO_3_, injecting up to two electrons each into the conduction band. Nevertheless, this $V_{O}^{..}$ concentration is insufficient to induce conductivity in the SrTiO_3_ channel.^[^ ^52^, ^53^ ^]^

Upon application of a voltage to the gate electrode, electrons accumulated beneath the gate insulator via the standard electrostatic‐gating effect, establishing a conductive FET channel (Figure 4b).^[^ ^54^ ^]^ Simultaneously, $V_{O}^{..}$ carrying up to +2*q* charge gradually drifted away from the gate‐insulator/SrTiO_3_ interface, resulting in a region with elevated $V_{O}^{..}$ concentration below the channel region. Consequently, the $V_{O}^{..}$ concentration decreased above the channel region just below the SrTiO_3_/insulator interface, prompting the generation of additional $V_{O}^{..}$ at the interface to counterbalance their decreased concentration. This build‐up of $V_{O}^{..}$ was accompanied by a progressive accumulation of the net electron concentration within the FET channel, thereby facilitating increased drain current.

Upon release of the gate voltage, electron depletion from the FET channel occurred promptly, leading to a decrease in drain current, typically below measurable levels (Figure 4c). Simultaneously, thermal diffusion drove $V_{O}^{..}$ migration toward the SrTiO_3_/insulator interface, gradually restoring their initial depth profile. Thus, $V_{O}^{..}$ drifted during voltage application and then diffused upon removal of the applied voltage. In principle, the accumulation of $V_{O}^{..}$ in the absence of electrostatic gating (i.e., without applied gate voltage) can induce a conductive FET channel if their concentration is raised above a threshold, making SrTiO_3_ metallic.

### 2.4 Finite Element Analysis of Oxygen Vacancy Drift–Diffusion

To further elucidate the dynamics of $V_{O}^{..}$ in the FET, we conducted a finite element analysis of oxygen drift–diffusion in SrTiO_3_ (detailed methodology is provided in the Experimental Section). In our simulations, the generation of $V_{O}^{..}$ at the SrTiO_3_ surface was assumed to be much faster than the diffusion of $V_{O}^{..}$, characterized by the diffusion coefficient *D*_vo_, within the SrTiO_3_ crystal. We assess the validity of this assumption in the subsequent discussion. The profiles of calculated oxygen vacancy concentration (*N*_VO_) and electron density distribution (*n*) for *D*_vo_ = 1 × 10^−11^ m^2^ s^−1^ are presented in Figure 4e,f, respectively, and the gate voltage (*V*_G_) ramping scheme is outlined in Figure 4d.

At *t* = 0.5 ms, corresponding to *V*_G_ = 0, only a low density of $V_{O}^{..}$ was present throughout the FET body (Figure 4e‐I). Upon ramping *V*_G_ to 5 V at *t* = 1.0 ms, the positive voltage caused $V_{O}^{..}$ to accumulate predominantly around the channel region, as shown in Figure 4e‐II. Notably, even after *V*_G_ settled at 5 V by *t* = 1.0 ms, *N*_VO_ continued to rise within the channel region and extended deeper into the FET body (Figure 4e‐III). This increase is a consequence of $V_{O}^{..}$ drift–diffusion, coupled with the continuous generation of $V_{O}^{..}$, which accumulated at the crystal surface. Although the gate voltage was applied between the laterally placed gate and source electrodes, electric fields that pointed downward and extended deep into the crystal were generated because of the large dielectric constant of SrTiO_3_, propelling the $V_{O}^{..}$ into the crystal (see Section S12, Supporting Information for the electric field distribution).

The electron distribution profile closely mirrors that of $V_{O}^{..}$. At *t* = 0.5 ms, electrons were completely depleted from the FET channel because of the band bending at the SrTiO_3_/insulator/gate electrode interface (Figure 4f‐I). Application of *V*_G_ caused electron density to increase around the channel region through the standard field‐effect gating mechanism (Figure 4f‐II). However, unlike in a conventional FET, the electron density continued to rise beyond *t* = 1.0 ms, spreading to a wider area around the channel region. This is because the $V_{O}^{..}$ that migrated in this region via drift–diffusion doped electrons into the conduction band of SrTiO_3_, increasing the carrier density and making the channel more conductive (Figure 4f‐III).

Figure 4h highlights the role of the diffusion coefficient of $V_{O}^{..}$ in determining the rate of *I*_D_ growth. Here, the time evolution of *I*_D_ was calculated for *D*_vo_ = 10^−9^, 10^−10^, 10^−11^, and 10^−12^ m^2^ s^−1^ (Figure 4h). *I*_D_ displayed gradual sustained growth after *t* = 1.0 ms, in line with the Drude model, i.e., *I*_D_∝*σ*∝*e* *µ*_e_ *n*, where *σ*, *e*, and *µ*_e_ are conductivity, elementary charge, and electron mobility, respectively. Our calculated *I*_D_ aligns well with the experimentally measured time evolution of *I*_D_ (Figure 4g) when *D*_vo_ = 10^−11^ m^2^ s^−1^. Thus, the drift–diffusion model effectively explains the leaky‐integration behavior of the SrTiO_3_ FET. Given that diffusion coefficients predominantly govern leaky‐integration behavior, variations of *V*_p_ and *V*_D_ exerted only minor effects on diffusion dynamics (see Section S6, Supporting Information for a discussion of the effect of *V*_D_ on $V_{O}^{..}$ drift–diffusion).

### 2.5 Functional Modeling of the Leaky‐Integration FET

So far, we have demonstrated the importance of long timescales in human‐interactive neuromorphic computing, highlighting the potential of leaky‐integration SrTiO_3_ FETs as components for constructing artificial neural networks with long timescales. To facilitate their practical application, it is crucial to develop a simple yet functional model for SrTiO_3_ FETs, because finite element modeling poses considerable computational challenges.

**Figure 5:**
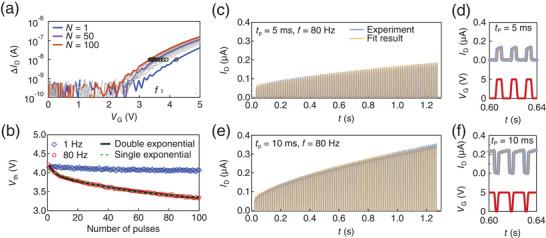
Functional modeling of leaky‐integration SrTiO_3_ FETs. a) Pulse‐dependent Δ*I*_D_ versus *V*_G_ curves of a SrTiO_3_ FET. Each curve corresponds to the data measured during different pulse cycles with *f* = 80 Hz, *t*_p_ = 5 ms, *V*_p_ = 5 V, and *V*_D_ = 0.5 V. Only the data for the first pulse (*N* = 1) and multiples of ten pulses are shown for clarity. The black pentagon indicates *V*_G_ when *I*_D_ exceeds 10 nA. b) Double‐exponential shift of the threshold voltage as a function of the number of pulses. The data at *f* = 80 Hz (red circles) were fitted to a double‐exponential function (black curve). For comparison, the fitting result using a single‐exponential function (green dashed curve) and data at *f* = 1 Hz (blue diamonds) are also shown. The sample size was 100. The probability value, statistical test, and significance symbol are not applicable. c,d,e,f) Time evolution of drain current during leaky integration fitted to the phenomenological drift–diffusion model Equations (1) and (2) when (c) *t*_p_ = 5 ms and (e) *t*_p_ = 10 ms. (d) and (f) are magnified views of (c) and (e), respectively.

To construct a quantitative model that characterizes the leaky‐integration behavior of SrTiO_3_ FETs, we first reexamined the leaky‐integration data in Figure 3b based on the idea that the drift–diffusion of $V_{O}^{..}$ donors alters the overall characteristics of SrTiO_3_ FETs. **Figure** **5**a plots pulse‐by‐pulse Δ*I*_D_ − *V*_G_ curves obtained by splitting the data in Figure 3b. Here, we defined $\Delta I_{D}=I_{D}+C\frac{dV_{G}}{dt}$ to exclude the displacement current contribution $-C\frac{dV_{G}}{dt}$ from *I*_D_ associated with the drain‐to‐gate and drain‐to‐source capacitance *C*. These data revealed a gradual shift of the Δ*I*_D_ − *V*_G_ curves to lower voltage after each pulse, thus resulting in an increase of Δ*I*_D_.

Next, we plotted the time evolution of the threshold voltage *V*_th_, defined as the gate voltage at which Δ*I*_D_ exceeded 10 nA (depicted as black pentagons in Figure 5a), as shown in Figure 5b. As the number of input pulses increased, *V*_th_ changed according to a double‐exponential decay. This exponential dependence on the *V*_th_ shift directly stems from the drift–diffusion phenomena discussed in the previous Section 2.3 and 2.4. Notably, the observed dependence of *V*_th_ on the number of input pulses cannot be adequately described by a single‐exponential decay, indicating the presence of multiple timescales within the physical system. The second timescale likely relates to that of oxygen vacancy generation at the interface between the SrTiO_3_ channel and gate insulator; its origin is discussed in Section 2.6. Practically, the existence of multiple timescales in neural networks such as reservoirs is useful because it allows signals with different rates to be handled within a single network. We also considered the time evolution of *V*_th_ for data collected at *f* = 1 Hz. Unlike the case when *f* = 80 Hz, *V*_th_ remained almost constant and exhibited minimal decay with increasing pulse number when *f* = 1 Hz. This frequency dependence suggests that *V*_th_ is influenced by the history of applied gate voltage, including the timing and width of gate pulses. This frequency‐dependent behavior originates from the balance between the electric field‐driven drift dynamics and thermal diffusion dynamics of $V_{O}^{..}$ in the SrTiO_3_ crystal. When the frequency is high, the $V_{O}^{..}$ accumulate around the FET channel driven by the applied gate‐voltage pulses before relaxing to their thermodynamic distribution. This results in the shift of *V*_th_ to smaller voltage because the accumulated $V_{O}^{..}$ behave as an electron donor to semiconducting SrTiO_3_. In contrast, when the frequency is low, *V*_th_ does not shift because the $V_{O}^{..}$ relax to their thermodynamic distribution between voltage pulses. Therefore, the *V*_th_ shift shown in Figure 5b is a direct consequence of the drift–diffusion dynamics explained in Section 2.3 and 2.4. Given that the shift of *V*_th_ primarily governs the leaky‐integration behavior of the FET, the weak dependence on *V*_p_ is desirable.

Considering that the shift of *V*_th_ depends on the history of the gate voltage *V*_G_(*t*), we redefined the threshold voltage as follows:

(1) $$\begin{array}{c} V_{\mathrm{th}}^{\prime }\left(t\right)=V_{0}-\int_{-\infty }^{t}V_{G}\left(x\right)\left(A_{1}\exp\left(-\frac{t-x}{\tau_{1}}\right)+A_{2}\exp\left(-\frac{t-x}{\tau_{2}}\right)\right)dx \end{array}$$

where *τ*_1_ and *τ*_2_ are time constants, the main parameters of leaky integration, and *A*_1_ and *A*_2_ are the corresponding contributions of these timescales to $V_{\mathrm{th}}^{\prime }\left(t\right)$, and *V*_0_ is the initial threshold voltage before applying gate voltage. Subsequently, the drain current can be expressed in terms of $V_{\mathrm{th}}^{\prime }\left(t\right)$ for subthreshold and linear regions of FET operation as follows^[^ ^55^ ^]^:

(2) $$\begin{array}{c} I_{D}=-C\frac{dV_{G}\left(t\right)}{dt}+\left\{\begin{array}{cc} I_{0}\exp\left(\left(V_{G}\left(t\right)-V_{\mathrm{th}}^{\prime }\left(t\right)\right)/V_{\mathrm{ss}}\right) & \left(V_{G}\left(t\right)\leq V_{\mathrm{th}}\left(t\right)\right)\\ I_{0}\left(1+z(V_{G}\left(t\right)-V_{\mathrm{th}}^{\prime }\left(t\right))\right) & \left(V_{G}\left(t\right)>V_{\mathrm{th}}\left(t\right)\right) \end{array}\right. \end{array}$$

where *C* is the combined gate and parasitic capacitance and *I*_0_, *V*_ss_, and *z* are phenomenological constants.

Equations (1) and (2) were used to describe the experimental data; the results are presented in Figure 5c–f. Our phenomenological model successfully captured the time evolution of *I*_D_ for the data obtained at both *t*_p_ = 5 ms and *t*_p_ = 10 ms using a single set of parameters: *τ*_1_ = 1.07 s, *τ*_2_ = 5.0 ms, *A*_1_ = 0.44 s^−1^, *A*_2_ = 15.7 s^−1^, *V*_0_ = 5.5 V, *I*_0_ = 108 nA, *V*_ss_ = 0.58 V, *z* = 1.9 V^−1^, and *C* = 0.88 pF. This model enables quantification of the long‐timescale leaky integration of SrTiO_3_ in the order of one second and lays the groundwork for simulating SrTiO_3_‐based neural networks.

### 2.6 Oxygen Vacancy Generation at the SrTiO_3_ Crystal Surface

In general, thermodynamics dictates that the distribution of $V_{O}^{..}$ depends on the electrostatic potential *V* and is given by $N_{\mathrm{vo}}=N_{\mathrm{vo}}^{\infty }\exp\left(-2eV/k_{B}T\right)$ at equilibrium, where $N_{\mathrm{vo}}^{\infty }$ is the *N*_vo_ value in a region far away from the active region of the device, *k*_B_ is the Boltzmann constant, and *T* is temperature.^[^ ^56^, ^57^ ^]^ Therefore, it might seem counterintuitive to observe an increase of *N*_vo_ with positive gate voltage, as shown in Figure 4e. However, under dynamic or steady‐state conditions (i.e., finite $V_{O}^{..}$ generation), *N*_vo_ deviates from the equilibrium distribution and depends on the concentration of $V_{O}^{..}$ at the SrTiO_3_ crystal surface. The drift–diffusion dynamics are governed by Fick's law ∂*N*_vo_/∂*t* = ∂(*D*_vo_∂*N*_vo_/∂*z*)/∂*z* with the boundary condition at the surface given by $k_{\mathrm{vo}}\cdot \left(N_{\mathrm{vo}}^{g}-N_{\mathrm{vo}}\right)=-{\left.D_{\mathrm{vo}}\partial N_{\mathrm{vo}}/\partial z\right|}_{\mathrm{surface}}$, where $N_{\mathrm{vo}}^{g}$ is the *N*_vo_ value at the external surface of SrTiO_3_, and *k*_vo_ is the oxygen‐exchange kinetic coefficient at the SrTiO_3_ surface.^[^ ^56^ ^]^ At the limit of fast $V_{O}^{..}$ generation at the SrTiO_3_ surface (*k*_vo_ ≫ *D*_vo_), the boundary condition simplifies to $N_{\mathrm{vo}}=N_{\mathrm{vo}}^{g}$. Under this condition, the increase of *N*_vo_ at a positive gate voltage can be attributed to the increased $V_{O}^{..}$ generation rate at the crystal surface, leading to an accumulation of $V_{O}^{..}$ in the region near the SrTiO_3_ surface.

In the case of SrTiO_3_ FETs, the source of $V_{O}^{..}$ is likely $V_{O}^{..}$ generated near the source and drain electrodes. These electrodes were fabricated by thermally depositing Ti metal directly onto SrTiO_3_; a process known to generate a relatively high concentration of $V_{O}^{..}$ near the surface through oxygen gettering and render SrTiO_3_ electrically conductive. This region effectively serves as a reservoir for $V_{O}^{..}$, meaning $V_{O}^{..}$ is readily available near the channel region, and thus making the condition *k*_vo_ ≫ *D*_vo_ relevant here. Additionally, electrical water splitting can occur at the SrTiO_3_ surface, which promotes $V_{O}^{..}$ generation and, consequently, nonvolatile switching of SrTiO_3_‐based FETs.^[^ ^58^ ^]^ Furthermore, recent studies suggest that electric field‐dependent proton transport can occur through oxides and polymers.^[^ ^59^, ^60^, ^61^, ^62^, ^63^ ^]^ Protons can induce $V_{O}^{..}$ formation when injected onto the surface of SrTiO_3_ through the gate insulator. While water and protons were not intentionally included in the FET structure, they may be present after lithography or in the environment. Although the value of *k*_vo_ in this device geometry is not precisely known, *k*_vo_ can be relatively large (resulting in shorter timescales) compared to *D*_vo_, especially when an external bias voltage is applied. Indeed, the time evolution of the threshold voltage (*V*_th_) followed a double‐exponential function, indicating multiple timescales in the system. The observation of multiple timescales possibly reflects the dynamics of $V_{O}^{..}$ generation combined with the slower drift–diffusion processes. If a single neuron element can exhibit both short and long timescales simultaneously, it holds the potential to enable the construction of neural networks with richer representation, thus forthcoming investigations in this direction are anticipated.^[^ ^64^ ^]^

## 3 Conclusion

We demonstrated that a FET made of the oxide semiconductor SrTiO_3_ can exhibit leaky‐integration behavior with long timescales. The operating mechanism of the SrTiO_3_ FET was underpinned by the drift–diffusion of $V_{O}^{..}$, which acted as mobile donors with a long timescale under the influence of an externally applied electric field. Unlike conventional CMOS devices with leaky integration, this device exhibited a distinct characteristic of conductance change evolution over long timescales, typically on the order of one second. The FET can operate with much lower energy consumption than that of conventional CMOS leaky‐integration devices because the leaky integration was induced solely through electrostatic means without net electrical currents or Joule heating (see Sections S9 and S14, Supporting Information for a discussion of energy consumption). Moreover, we found that the timescales of the leaky integration were insensitive to the channel size of the transistor. This invariability of leaky‐integration timescale was caused by its drift–diffusion mechanism and will aid device miniaturization, contributing to further decreasing energy consumption without affecting the timescale (see Section S9, Supporting Information for a discussion of scalability). Timescale may be adjusted by, for example, engineering the electrostatic profile through back‐gating. These general concepts can be applied to other oxides to realize long timescales in miniaturized devices.^[^ ^65^ ^]^ We also highlighted the critical role of timescales in devices used in artificial neural networks, particularly in the context of energy‐efficient neuromorphic computing. A long timescale is indispensable for tasks that process human interactive signals. Our findings not only contribute to advancing the field of neuromorphic computing but also offer insights into harnessing emergent phenomena in oxide semiconductors to realize novel computing paradigms.

## 4 Experimental Section

### Fabrication of the SrTiO_3_ FET

The SrTiO_3_ FET was fabricated using standard photolithography.^[^ ^50^, ^51^ ^]^ First, a 2.7 nm‐thick Parylene C film deposited on a commercial SrTiO_3_ substrate (Shinkosha, Co.) by chemical vapor deposition was partially etched by UV‐ozone irradiation to provide contact areas for the source and drain electrodes, which were subsequently formed by thermal deposition of Ti. A 2.8 nm‐thick Parylene C film was deposited on the entire substrate by chemical vapor deposition to form the gate dielectric followed by a 20 nm‐thick HfO_*x*_ layer formed by atomic layer deposition. Next, the double‐layer Parylene C film was removed from the bonding pad regions by UV‐ozone irradiation. A thick SiO_*x*_ layer was deposited on the gate bonding pad regions by radio‐frequency sputtering to ensure electrical isolation. Deposition of thick Ti/Au bonding pads and Ti/Au gate electrodes on the channel regions completed the FET fabrication process. The channel length and width were 2 and 8 µm, respectively.

### Characterization of the SrTiO_3_ FET

The SrTiO_3_ FET was characterized by applying a time‐dependent voltage to the drain and gate electrodes using a function generator (WF1948, NF Circuit Design, Co.) and measuring the current flowing through the drain electrode using a device current waveform analyzer (CX3324A, Keysight Technologies, Inc.).

### Finite Element Analysis of Drift–Diffusion Dynamics

Distributions of electrons and $V_{O}^{..}$ were modeled by the finite element method using COMSOL software (COMSOL, Inc.) using the semiconductor and electrochemistry modules with the quasi‐Fermi energy, electric potential, and oxygen vacancy concentration as model variables. For simplicity, the calculation was performed in a 2D geometry of 10 × 10 µm and all quantities were assumed to be uniform in the depth direction. The model assumed contributions only from the conduction band of SrTiO_3_ and that the oxygen vacancy donor was fully activated. Electron transport was calculated based on the standard Drude model with a constant relaxation time, and other effects, such as carrier trapping, were not included. The background concentration of $V_{O}^{..}$ was set to 10^15^ cm^−3^. The boundary condition for oxygen vacancy concentration was *N*_vo_ = 10^15^ cm^−3^ for both the boundaries inside SrTiO_3_ and at the crystal surface, assuming that the oxygen‐vacancy generation rate at the surface was much faster than their diffusion rate.

### Handwriting Anomaly Detection

Handwriting anomaly detection was demonstrated by numerically simulating reservoir dynamics using MATLAB software (Mathworks, Inc.) on a personal computer. The trajectory data were first stored in a data file and later fed into the simulation program. The position of a pen was recorded at a time interval of 10 ms as each research participant moved the pen on the input device. The positional coordinates in Figure 2b are in units of pixels scaled by a factor of 0.01. The reservoir was constructed as a randomly connected spiking neural network. Leaky‐integrate‐and‐fire neurons were used as the neuron model. The number of neurons used in the reservoir was 256. The detection was performed by first feeding the handwriting trajectory of person A as training data and recording all the states of constituting neurons. Next, the handwriting trajectory of a different person B (testing data, anomalous input) was input and the resulting neurons states were recorded. This process was repeated for person A's different trajectory (testing data, standard input). To quantify degrees of anomaly, the Mahalanobis distance of reservoir states, *S*_A_, from the neuron states using a time step of 1 ms were calculated.^[^ ^49^ ^]^ The value of *S*_A_ became large only when the neuron states differed considerably from the states during the training phase. Two kinds of reservoirs: one with long time constants (*τ*_n_ = 1.07 s) and the other with short time constants (*τ*_n_ = 10 µs) were simulated.

The dynamics of neural networks were simulated step by step with time intervals of 0.1 ms and 0.1 µs for the long‐timescale (*τ*_n_ = 1.07 s) and short‐timescale (*τ*_n_ = 10 µs) reservoirs, respectively. To match the time intervals of the trajectory data with the simulation, the same values were fed into the neural network 100 times (*τ*_n_ = 1.07 s) and 100 000 times (*τ*_n_ = 10 µs) per data point before proceeding to the next data point. It took ≈3.8 and 3800 s for the long‐timescale and short‐timescale reservoirs, respectively, to simulate the 1‐s equivalent of the neural network dynamics. The long simulation time for the short‐timescale reservoir was caused by the large number of simulation steps required to minimize the time discretization error. Details of the simulation are outlined in Section S4 (Supporting Information). See Section S11 (Supporting Information) for further discussion of the readout functions of the reservoir.

The handwriting trajectory data in Figure 2b were acquired using a tablet device after informed written consent from all participants. This research was conducted with the approval of the ethics board, Life Science Experiment Management Office, National Institute of Advanced Industrial Science and Technology, Approval No. 20221237C.

### Statistical Analysis

In Figure 5b, the data fitting was performed by the Levenberg–Marquardt least‐squares method using Igor Pro software (WaveMetrics, Inc.). The sample size was 100 and the data points were obtained from the *I*_D_ − *V*_G_ curves shown in Figure 5a. No further pre‐processing of the data was conducted. Single‐ and double‐exponential functions of the form *y*_0_ + *A*exp (− *γ* *x*) and *y*_0_ + *A*_1_exp (− *γ*_1_ *x*) + *A*_2_exp (− *γ*_2_ *x*), respectively, were used as the fitting functions. The fittings yielded the coefficients *y*_0_ = 3.26 ± 0.017 V, *A* = 0.80 ± 0.014 V, *γ* = 0.021 ± 0.0011 for the single‐exponential function and *y*_0_ = 3.09 ± 0.024 V, *A*_1_ = 0.87 ± 0.018 V, *γ*_1_ = 0.0127 ± 0.00067, *A*_2_ = 0.247 ± 0.0098 V, *γ*_2_ = 0.30 ± 0.025 for the double‐exponential function. No statistical tests were performed in this study.

## Conflict of Interest

The authors declare no conflict of interest.

## Supporting information

Supporting Information

## Data Availability

The data that support the findings of this study are available from the corresponding author on reasonable request.
