# Supplementary material for: Taming Prolonged Ionic Drift–Diffusion Dynamics for Brain‐Inspired Computation
Source: Adv Mater. 2024 Nov 27;37(3):2407326. doi: 10.1002/adma.202407326 (PMC11756045; doi:10.1002/adma.202407326)
Supplement: Supplementary file 1 — Supporting Information [file ADMA-37-2407326-s001.pdf]

ADVANCED MATERIALS

Supporting Information

for *Adv. Mater.*, DOI 10.1002/adma.202407326

Taming Prolonged Ionic Drift–Diffusion Dynamics for Brain-Inspired Computation

Hisashi Inoue, Hiroto Tamura, Ai Kitoh, Xiangyu Chen, Zolboo Byambadorj, Takeaki Yajima, Yasushi Hotta, Tetsuya Iizuka, Gouhei Tanaka and Isao H. Inoue**

Supporting information for:

Taming Prolonged Ionic Drift-Diffusion Dynamics for Brain-Inspired Computation

Hisashi Inoue Hiroto Tamura Ai Kitoh Xiangyu Chen Zolboo Byambadorj Takeaki Yajima Yasushi Hotta Tetsuya Iizuka Gouhei Tanaka Isao H. Inoue**

H. Inoue, A. Kitoh, I. H. Inoue

National Institute of Advanced Industrial Science and Technology (AIST), Tsukuba 305-8565, Japan Emails: hisashi.inoue@aist.go.jp, i.inoue@aist.go.jp

H. Tamura

Graduate Schools for Law and Politics, The University of Tokyo, Tokyo 113-0033, Japan

H. Tamura, G. Tanaka

International Research Center for Neurointelligence (IRCN), The University of Tokyo, Tokyo 113-0033, Japan

X. Chen, Z. Byambadorj, T. Iizuka

Systems Design Lab., School of Engineering, The University of Tokyo, Tokyo 113-0032, Japan

T. Yajima

Graduate School of Information Science and Electrical Engineering, Kyushu University, Fukuoka 819-0395, Japan

Y. Hotta

Department of Engineering, University of Hyogo, Hyogo 671-2280, Japan

G. Tanaka

Department of Computer Science, Nagoya Institute of Technology, Nagoya 466-8555, Japan

1 Static characteristics of $SrTiO_3$ FET

Figure S1a shows the quasi-static $I_D - V_G$ characteristic of the $SrTiO_3$ FET. The gate leak I_D as a function of V_G is also shown in Figure S1b. The $SrTiO_3$ FET shows favorable FET characteristics with an on-to-off ratio over 10^6 and minimum gate leakage below 30 pA. The data shows hysteresis due to oxygen vacancy drift-diffusion in the $SrTiO_3$ FET during the measurement cycle. One measurement cycle takes about 100 seconds. We used a semiconductor parameter analyzer (4155C, Agilent, Inc.) to conduct the measurement.

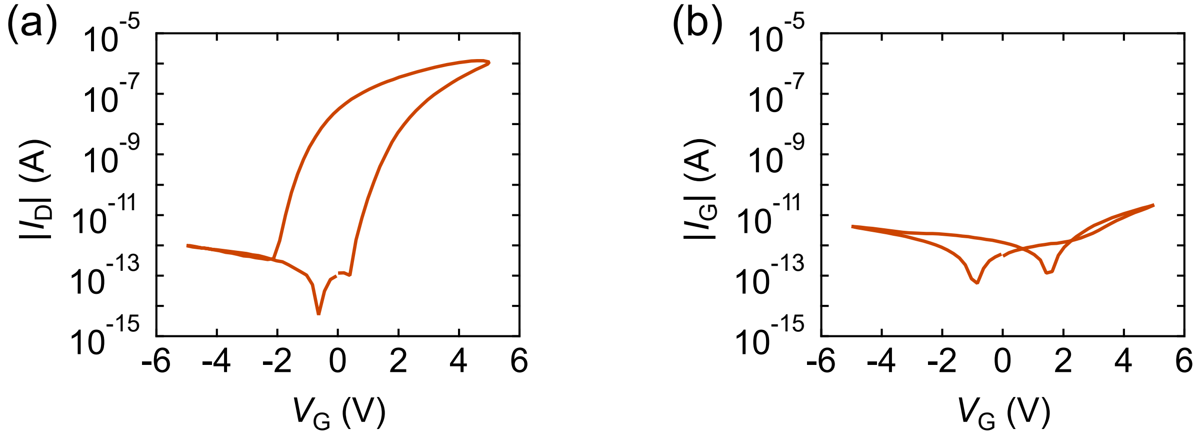

Figure S1: a) Quasi-static I_D versus V_G curve, and b) quasi-static I_G versus V_G curve.

2 Leaky-integration behavior of $SrTiO_3$ FET

In Figure 3b,c of the main text, we have demonstrated the leaky-integration behavior of $SrTiO_3$ for the pulse width $t_p = 5$ ms, pulse amplitude $V_p = 5$ V, and drain voltage $V_D = 0.5$ V. Figure S2 presents the leaky integration behavior for different variations of these parameters. In each case, the $SrTiO_3$ FET shows qualitatively the same behavior, with the overall amplitude of I_D remaining almost the same at low frequencies but gradually increasing at higher frequencies. Therefore, these results clearly illustrate the leaky-integration function of the $SrTiO_3$ FET. Furthermore, the leaky-integration rates presented in Figure 3e-g of the main text were extracted from a dataset that includes some of the results shown in Figure S2.

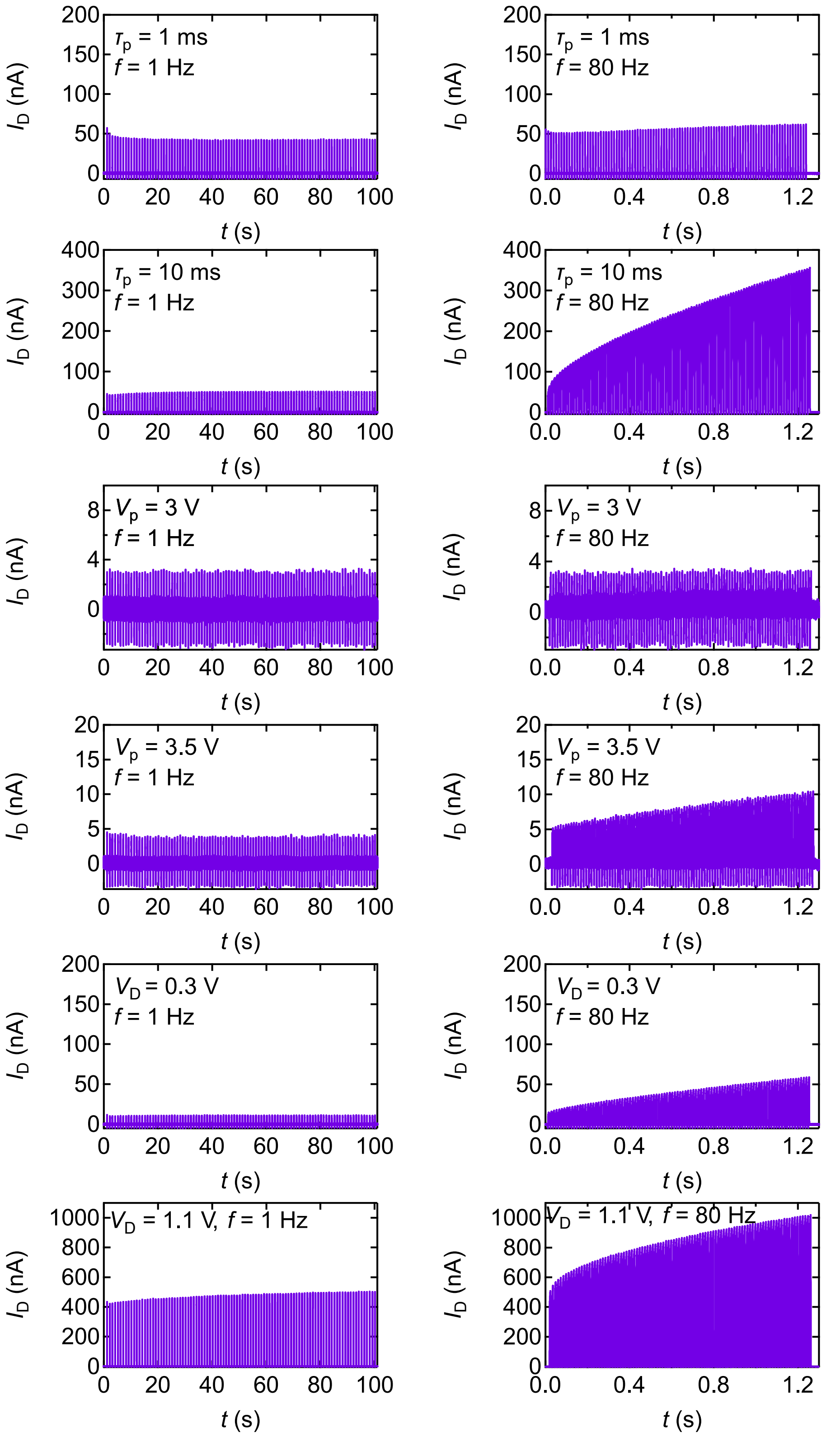

Figure S2: Leaky-integration behavior of $SrTiO_3$ FET for a variety of operation conditions; $\tau_p = 5$ ms, $V_p = 5$ V, and $V_D = 0.5$ V, unless otherwise noted.

3 CMOS-based leaky-integrate neurons

To compare with the long-timescale leaky integration of the $SrTiO_3$ FET, we have developed a CMOS-based leaky-integrate neuron, as shown in Figure S3a. It was designed using a differential amplifier, pseudo-resistors (PR), and a capacitor (C_{mem}). The device was implemented as ASIC manufactured using the TSMC 65 nm CMOS process. The advantage of using PR is two-fold: (a) PR can achieve relatively large resistance up to a few hundred MΩ, and (b) the resistance can be modified by external bias voltages [1]. Figure S3b and S3c show the leaky-integration operation of a CMOS-based neuron. Note that the polarity of V_{out} was reversed because of the construction shown in Figure S3a. The output voltage V_{out} was measured by an oscilloscope via an integrated source follower. When a pulse is given as input, V_{out} gradually decreases while V_{in} is on (integration). However, V_{out} gradually recovers to the original level when V_{in} is turned off (leak). When a series of pulses is given as input, V_{out} increases after every pulse, resulting in an overall increase of V_{in} after 50 pulses. These results demonstrate a leaky-integration operation similar to the $SrTiO_3$ FET, but the timescales of leaky-integration are different. Figure S3d summarizes leaky-integration rate $V_{out}(100\text{th pulse})/V_{out}(1\text{st pulse})$ as a function of inverse pulse frequency f^{-1}. This graph shows that the leaky-integration rate approaches unity at $f^{-1} \sim 10$ μs. Interpreting this value as the timescale of leaky integration, it is orders of magnitude smaller than the timescales of $SrTiO_3$ FET. Based on these results, we used the timescale $\tau_n = 10$ μs for simulating CMOS-based neurons in Figure 2d of the main text.

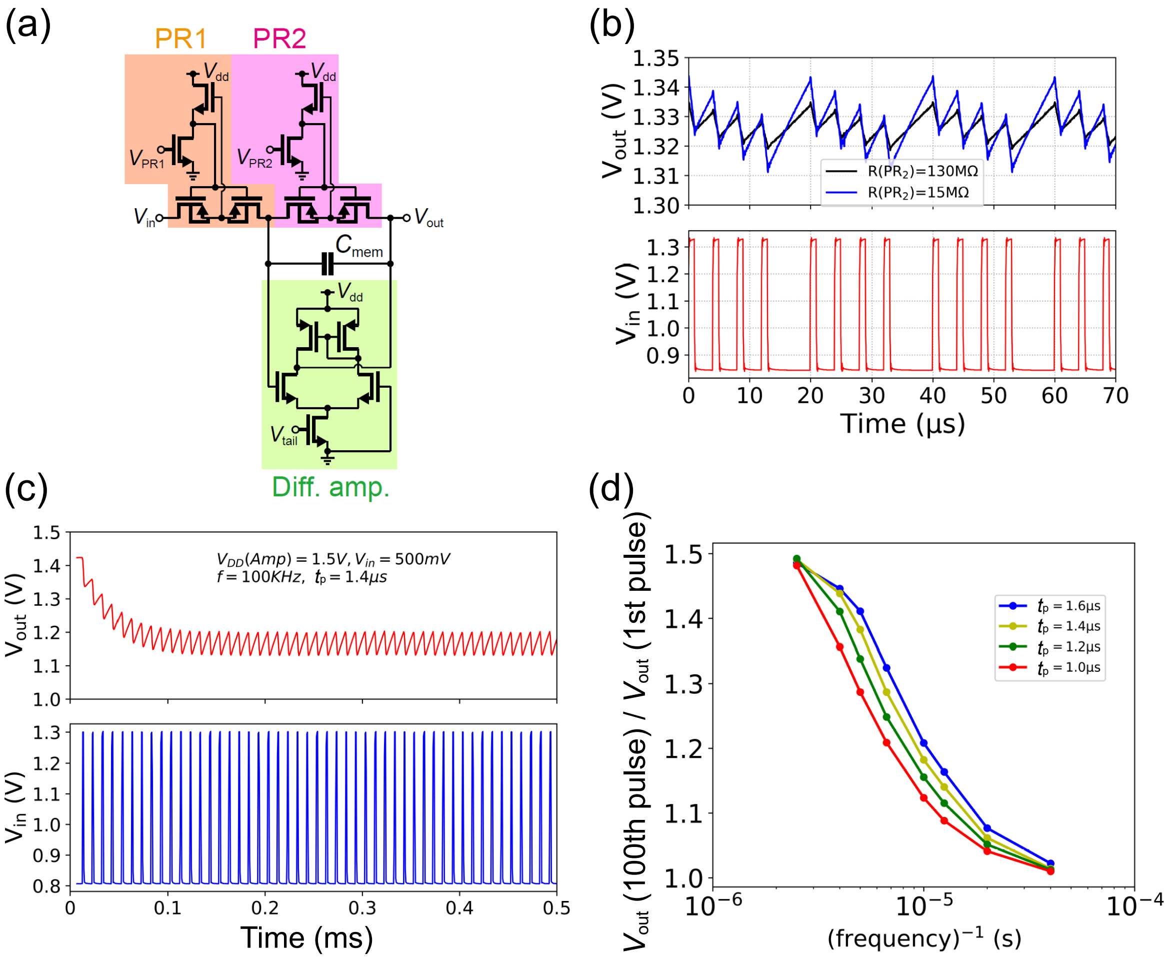

Figure S3: a) Schematic diagram of the CMOS-based leaky-integrate neuron. b) Leaky-integrate operation of the CMOS-based neuron. c) The time evolution of V_{out} when a train of pulses is inputted to V_{in}. d) Leaky-integration rates as a function of inverse frequency for various widths of the input pulses.

4 Handwriting anomaly detection

This section outlines the process of detecting handwriting anomalies using spiking-neural-network-based reservoir computing. Reservoirs can be constructed using randomly connected neural elements. Leaky-integrate-and-fire neurons and dynamic synapses connected in series were used as the neural element. The leaky-integrate-and-fire neuron transforms a continuous-valued input into a series of spikes, and the dynamic synapse converts these spikes into a continuous-valued output. The output signal of a neuron $s(t)$ in the reservoir is given by

$$s(t) = \begin{cases} 1 & ((t-t_\mathrm{s}) < T_\mathrm{s}) \\ 0 & ((t-t_\mathrm{s}) \geq T_\mathrm{s}), \end{cases} \tag{1}$$

where T_s is the duration of the spike, and t_s is the time of the last spike. The spike is generated when the neuron's membrane potential v_m exceeds the threshold value v_c. The equations that govern v_m are as follows:

$$\begin{cases} \tau_\mathrm{n} \frac{dv_\mathrm{m}(t)}{dt} = -v_\mathrm{m}(t) + i(t) & ((t-t_\mathrm{s}) \geq T_\mathrm{r}) \\ v_\mathrm{m}(t) = 0 & ((t-t_\mathrm{s}) < T_\mathrm{r}). \end{cases} \tag{2}$$

Here, τ_n is the time constant of the neuron, T_r is the refractory period, and $i(t)$ is the input to the neuron. The time evolution of output from the dynamic synapse $r(t)$ is described by an exponential decay after a spike input as follows:

$$\tau_\mathrm{s} \frac{dr(t)}{dt} = -r(t) + \frac{s(t)}{T_\mathrm{s}}. \tag{3}$$

Here, τ_s is the time scale of the synapse and is assumed to have the same value as τ_n. The reservoir dynamics are expressed using the collection vector of $r(t)$ of all neural elements in the reservoir $\boldsymbol{r}(t) \in \mathbb{R}^N$, called response trajectory [2]. Combined with Eqs. (1), (2), and (3), the N-dimensional vector $\boldsymbol{i}(t) \in \mathbb{R}^N$ that consists of the input currents $i(t)$ to all N neurons are given by the following equation:

$$\boldsymbol{i}(t) = q(gW\boldsymbol{r}(t)^\top + H\boldsymbol{i}_\mathrm{e}(t)^\top) + \boldsymbol{i}_\mathrm{b}. \tag{4}$$

Here, $\boldsymbol{i}_\mathrm{e} = (x(t), y(t))$ represents the external input to the reservoir (i.e. coordinates of the pen), $\boldsymbol{i}_\mathrm{b} \in \mathbb{R}^N$ represents the constant bias input to the neurons, $W \in \{-2s^2, -s^2, 0, s^2, 2s^2\}^{N\times N}$ represents the weight matrix describing the interconnection between neural elements in the reservoir, $H \in \{-0.5, 0, 0.5\}^{N\times 2}$ represents the input connection matrix, s^2 is the variance of the values in W, and q and g are the external and internal signal gains of the reservoir,

respectively.

The calculation was performed using a time-discretized form with the interval Δt. The equations used for updating neuronal and synaptic states in the reservoir are described as follows:

$$\begin{cases} v_\mathrm{m}(t+\Delta t) = v_\mathrm{m}(t) - \frac{1}{\tau_\mathrm{n}}\left[v_\mathrm{m}(t) - i(t)\right]\Delta t & ((t-t_\mathrm{s}) \geq T_\mathrm{r}) \\ v_\mathrm{m}(t) = 0 & ((t-t_\mathrm{s}) < T_\mathrm{r}), \end{cases} \tag{5}$$

$$r(t+\Delta t) = r(t) - \frac{1}{\tau_\mathrm{s}}\left[r(t) - \frac{s(t)}{T_\mathrm{s}}\right]\Delta t. \tag{6}$$

Handwriting anomaly detection was performed by first feeding the handwriting trajectory of person A (standard input) into the reservoir and recording $\boldsymbol{r}(t)$. Then, this was compared to $\boldsymbol{r}(t)$ when the handwriting trajectory of a different person B (anomalous input) was input. The reservoir converts the input signal to the response trajectory, whose geometry in the N-dimensional space characterizes the temporal feature of the input trajectory. It can be shown [2] that the response trajectories generated by similar input (i.e., by person A) are confined to a single linear subspace of the N-dimensional feature space, whereas those generated by dissimilar input (i.e., by person B) deviate from the subspace. The distance from the subspace is quantified by the anomaly score called Mahalanobis distance of reservoir states $S_\mathrm{A} = \boldsymbol{r}(t)^\top P\boldsymbol{r}(t)$, with the precision matrix $P = (\Sigma_0 + \alpha I)^{-1}$, which is related to the covariance matrix $\Sigma_0 = \Sigma_t \boldsymbol{r}(t)\boldsymbol{r}(t)^\top$ (sum is taken over all or some of the training data points), α is a regularization parameter (set to 0.01 in the current study), and I is the identity matrix. Therefore, we expect a small S_A for the handwriting input from the same person but a large S_A for input from a different person (see Supplementary Section 11 for further discussion on the readout functions of the reservoir).

We have simulated two kinds of reservoirs: one with long-time constants ($\tau_\mathrm{n} = \tau_\mathrm{s} = 1.07$ s, $T_\mathrm{r} = 1$ ms, $T_\mathrm{s} = \Delta t = 0.1$ ms) and the other with short-time constants ($\tau_\mathrm{n} = \tau_\mathrm{s} = 10$ μs, $T_\mathrm{r} = 1$ μs, $T_\mathrm{s} = \Delta t = 0.1$ μs). q and g were adjusted to regulate the firing rate of neurons and to adjust the magnitude of $|r(t)| \lesssim 1$ ($q = 20000, g = 0.001$ for the long-timescale reservoir and $q = 100, g = 0.125$ for the short-timescale reservoir). We set the value of W so that its elements followed the normal distribution with the mean and variance (s^2) equal to 0 and $1.25/N$, respectively. Only 25 % of the elements in H had non-zero values drawn from the uniform distribution with a

mean value of 0. The precision matrix P was updated online during the training using the formula

$$P(t+\Delta\tau) = P(t) - \frac{P(t)\boldsymbol{r}(t)\boldsymbol{r}(t)^\top P(t)}{1+\boldsymbol{r}(t)^\top P(t)\boldsymbol{r}(t)}, \tag{7}$$

$$P(0) = \frac{I}{\alpha} \tag{8}$$

instead of its definition, where $\Delta\tau$ is a time interval of the update, and $\Delta\tau = 1$ ms was used for both the long-timescale and short-timescale reservoirs.

5 Band diagrams of the leaky-integrating $SrTiO_3$ FET

The electron distribution presented in the main Figure 4 can be understood from the perspective of the band diagrams of the leaky-integrating $SrTiO_3$ FET. The cross-sectional band diagram of the $SrTiO_3$ field effect transistor at the middle point of the channel is calculated by the finite element analysis and is presented in Figure S4. E_{vac}, E_c, E_v, and E_F stand for the vacuum level, conduction band bottom energy, valence band top energy, and quasi-Fermi level, respectively. Note the different horizontal axis scales for $z < 0$ and $z \geq 0$ in Figure S4b, c, and d for visibility. Band parameters used in the band diagram are reported in [3, 4, 5, 6]. Since the electron affinity of Parylene C is not known, the center of the Parylene C band is assumed to be aligned with the center of the HfO_x band.

At $t = 0.5$ ms with $V_G = 0$ V (Figure S4b), the $SrTiO_3$ band is partially depleted by electron transfer from $SrTiO_3$ to the Au gate electrode due to the large work function of Au. When $V_G = 5$ V is applied at $t = 1.0$ ms (Figure S4c), the $SrTiO_3$ band bends downward towards the $SrTiO_3$/Parylene C interface, and electrons are accumulated around this region. The non-uniform E_F within $SrTiO_3$ reflects the electron migration that counteracts the oxygen vacancy migration into the $SrTiO_3$ substrate. At $t = 10.0$ ms while keeping $V_G = 5$ V (Figure S4d), the band bending around the interface becomes shallower. As a result, E_c approaches E_F on a broader region than at $t = 1.0$ ms, indicating more spread electron distribution deep into the $SrTiO_3$ substrate. Therefore, the time evolution of electron distribution presented in the main text Figure 4f is a direct consequence of the band structure change described in this section and is consistent with the phenomenological description given in the main text Section 2.3.

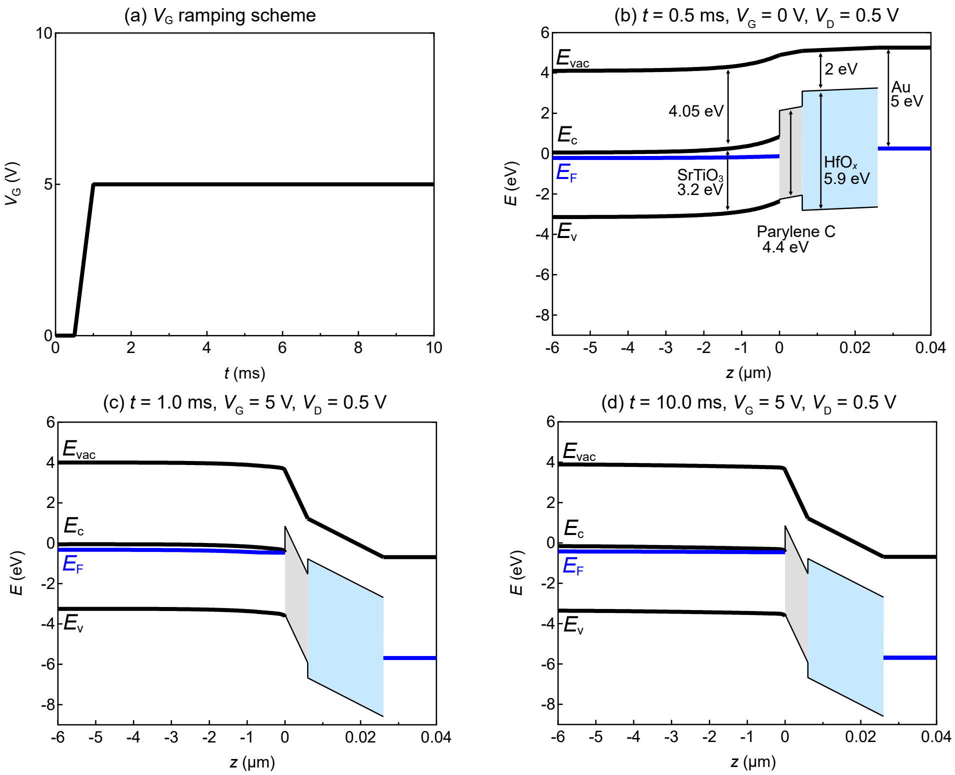

Figure S4: a) The V_G ramping scheme used in the calculation. b-d) Band structure of the leaky-integrating $SrTiO_3$ FET (b) at $t = 0.5$ ms before V_G application, (c) at $t = 1.0$ ms just after application of V_G, and (d) at $t = 10.0$ ms while keeping $V_G = 5$ V. Note the different horizontal axis scales in (b)-(d) for $z < 0$ and $z \geq 0$ for visibility.

6 Effect of drain voltage on the oxygen vacancy drift-diffusion

In the main text Figure 3g, V_D dependence of the leaky-integration ratio $I_D(N = 100)/I_D(N = 1)$ is presented. We see that $I_D(N = 100)/I_D(N = 1)$ decreases as V_D increases at high frequencies $f = 140$ Hz ($f^{-1} \sim 0.007$ s). This V_D dependence comes from the fact that the application of drain voltage works in addition to the effect of V_G to drive the oxygen vacancy drift-diffusion by generating a potential gradient in the lateral direction of the transistor device. In other words, the initial oxygen vacancy concentration before applying the V_G pulse depends on V_D. When $V_D = 1.1$ V, a relatively large amount of oxygen vacancies are generated around the drain electrode by the positive potential of the electrode, and they migrate towards the channel region of FET (Figure S5a). As a result, I_D is large from the initial V_G pulse, and the change of I_D during the subsequent V_G pulses is marginal because oxygen vacancies are saturated around the channel region (Figure S5b). In contrast, when V_D is small ($V_D = 0.3$ V), the initial oxygen vacancy concentration is small (Figure S5c), and I_D increases by a significant amount during the subsequent V_G pulses (Figure S5d).

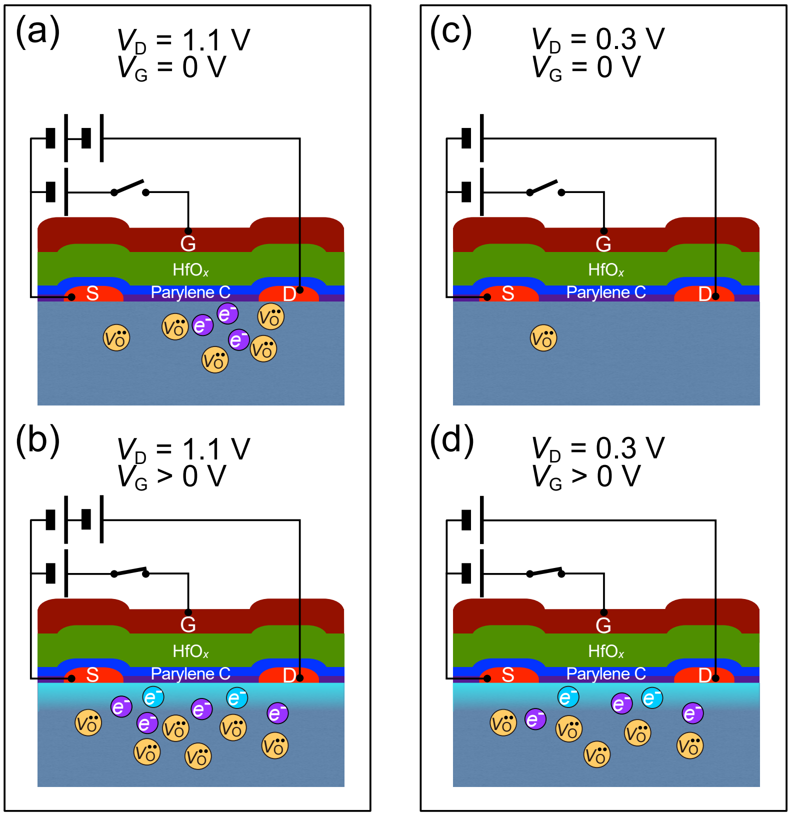

Figure S5: a) Schematic oxygen vacancies distribution in $SrTiO_3$ substrate when $V_D = 1.1$ V and $V_G = 0$ V. Relatively large concentrations of oxygen vacancies are generated before the application of V_G. b) Schematic oxygen vacancies distribution in $SrTiO_3$ substrate when $V_D = 1.1$ V and $V_G > 0$ V. The oxygen vacancy concentration increases only slightly because the oxygen vacancies are saturated. c-d) Same as (a) and (b), but for the case $V_D = 0.3$ V. The oxygen vacancy concentration increases more significantly from (c) to (d) than from (a) to (b) owing to the small initial oxygen vacancy concentration as depicted in (c).

7 Characterization of additional leaky-integrating $SrTiO_3$ FETs

Figure S6, S7, S8, and S9 present the characterization results of four additional leaky-integrating $SrTiO_3$ FETs. The channel lengths L and widths W of the devices are $L = 2$ μm and $W = 8$ μm for Figure S6, $L = 4$ μm and $W = 16$ μm for Figure S7, $L = 4$ μm and $W = 16$ μm for Figure S8, and $L = 9$ μm and $W = 36$ μm for Figure S9. From panels a, b, and c of Figure S6, S7, S8 and S9, the overall trends of the frequency dependence on the leaky-integration ratio $I_D(N = 100)/I_D(N = 1)$ for various widths of the input gate voltage pulse, the height of the pulse, and V_D are consistent among these four devices and the device presented in the main text Figure 3. The actual values of $I_D(N = 100)/I_D(N = 1)$ vary from device to device by a factor of about 2. We expect such variation in device characteristics to be advantageous in artificial neural networks for information processing because it helps neural activity adapt to a variety of input signals to process information.

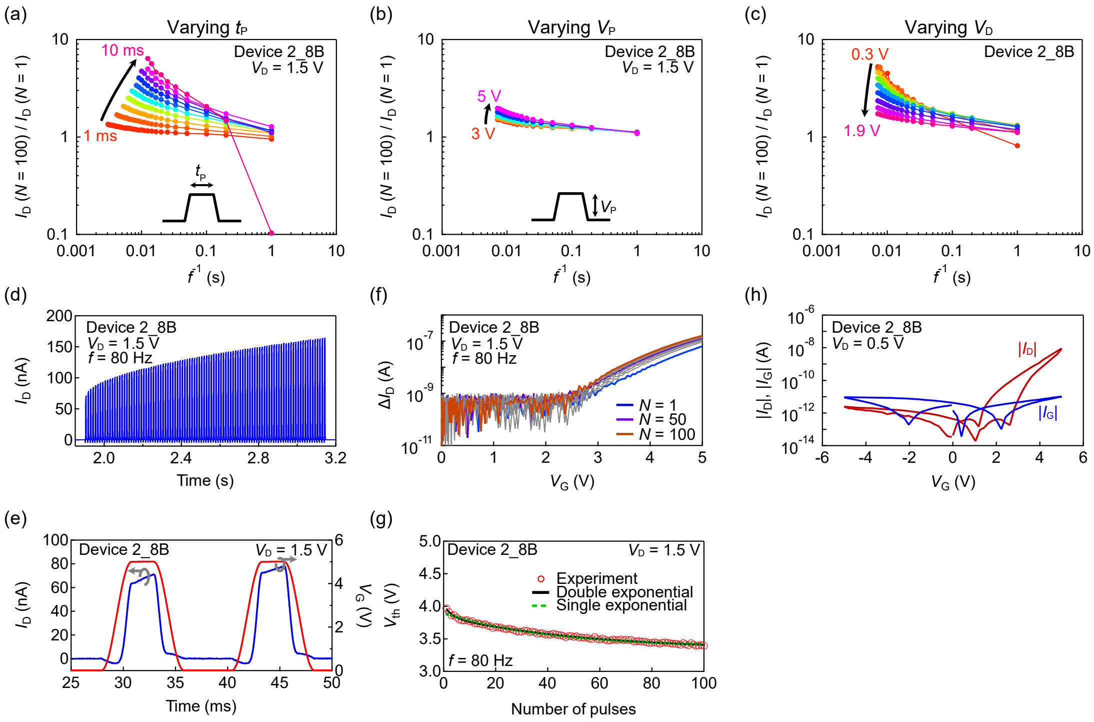

Figure S6: Characterization results of a different Device 2_8B with $L = 2$ μm and $W = 8$ μm. a-c) Frequency dependence of the leaky-integration ratio $I_D(N = 100)/I_D(N = 1)$ for (a) various pulse widths t_p, (b) pulse height V_p, and (c) drain voltages V_D. d) Temporal response of Device 2_8B to the gate voltage pulses. e) Magnified view of (d). f) Pulse-by-pulse $\Delta I_D - V_G$ curves during leaky integration for the different numbers of the pulses (N). ΔI_D is defined as I_D with the displacement current contribution $-C\frac{dV_G}{dt}$ subtracted. g) Shift of the threshold voltage V_{th} extracted from (f) during leaky integration. h) Quasi-static $I_D - V_G$ and $I_G - V_G$ curves of Device 2_8B.

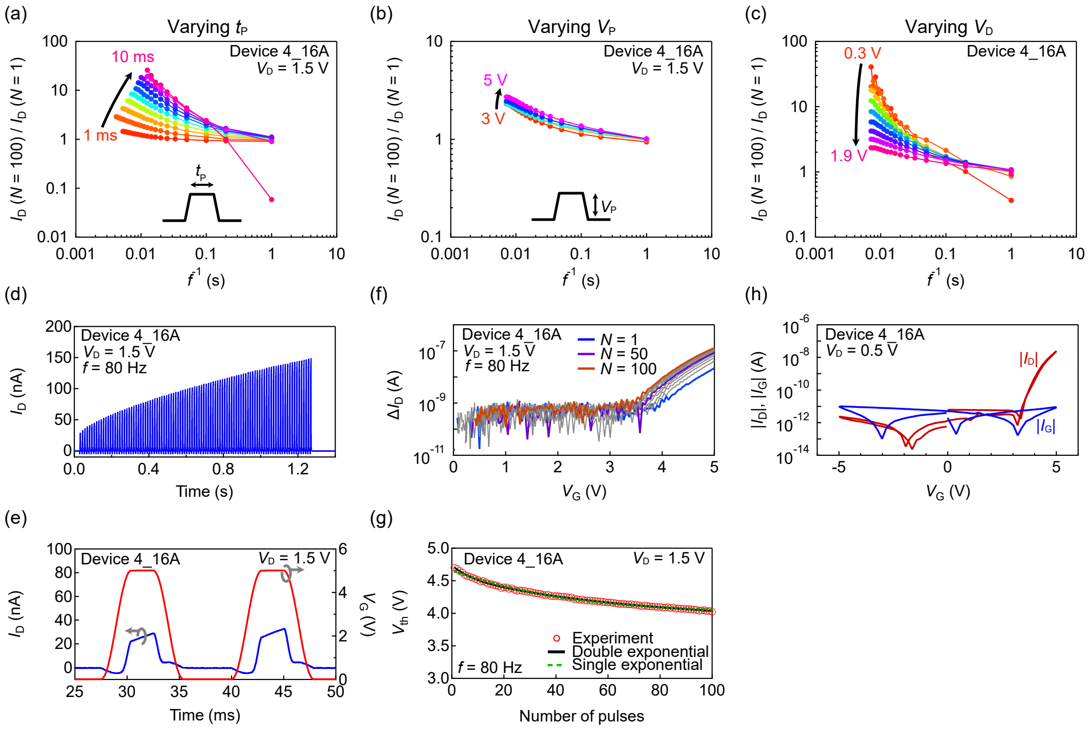

Figure S7: Characterization results of a different Device 4_16A with $L = 4$ μm and $W = 16$ μm. a-c) Frequency dependence of the leaky-integration ratio $I_D(N = 100)/I_D(N = 1)$ for (a) various pulse widths t_p, (b) pulse height V_p, and (c) drain voltages V_D. d) Temporal response of Device 4_16A to the gate voltage pulses. e) Magnified view of (d). f) Pulse-by-pulse $\Delta I_D - V_G$ curves during leaky integration for the different numbers of the pulses (N). ΔI_D is defined as I_D with the displacement current contribution $-C\frac{dV_G}{dt}$ subtracted. g) Shift of the threshold voltage V_{th} extracted from (f) during leaky integration. h) Quasi-static $I_D - V_G$ and $I_G - V_G$ curves of Device 4_16A.

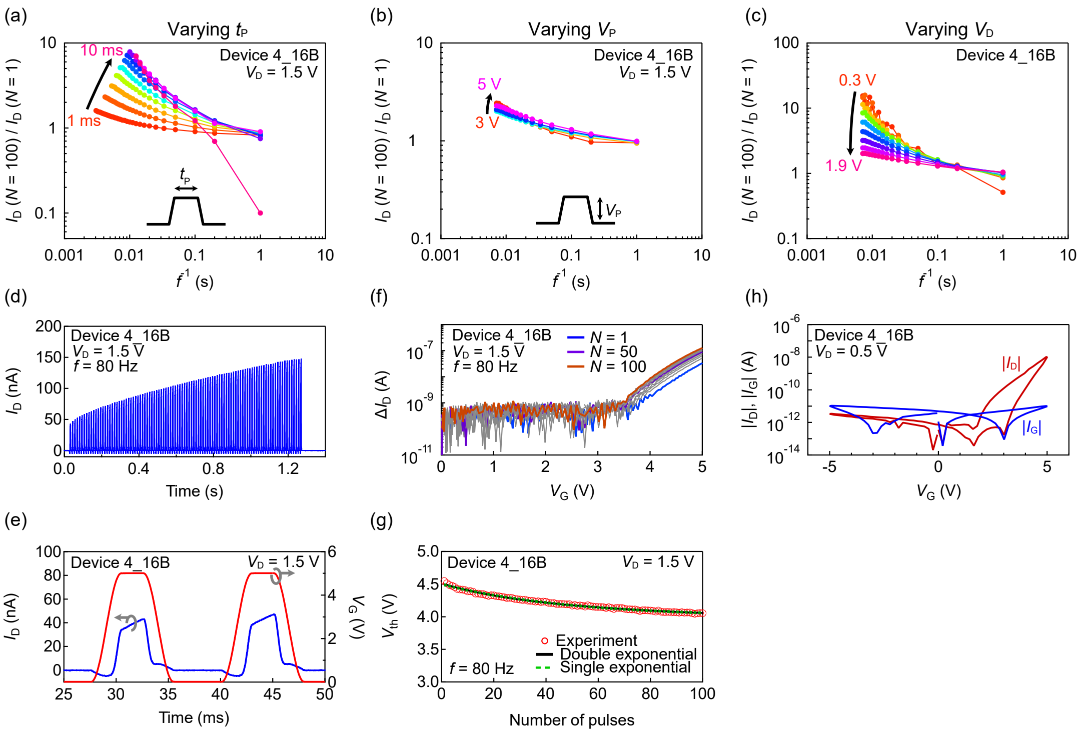

Figure S8: Characterization results of a different Device 4_16B with $L = 4$ μm and $W = 16$ μm. a-c) Frequency dependence of the leaky-integration ratio $I_D(N = 100)/I_D(N = 1)$ for (a) various pulse widths t_p, (b) pulse height V_p, and (c) drain voltages V_D. d) Temporal response of Device 4_16B to the gate voltage pulses. e) Magnified view of (d). f) Pulse-by-pulse $\Delta I_D - V_G$ curves during leaky integration for the different numbers of the pulses (N). ΔI_D is defined as I_D with the displacement current contribution $-C\frac{dV_G}{dt}$ subtracted. g) Shift of the threshold voltage V_{th} extracted from (f) during leaky integration. h) Quasi-static $I_D - V_G$ and $I_G - V_G$ curves of Device 4_16B.

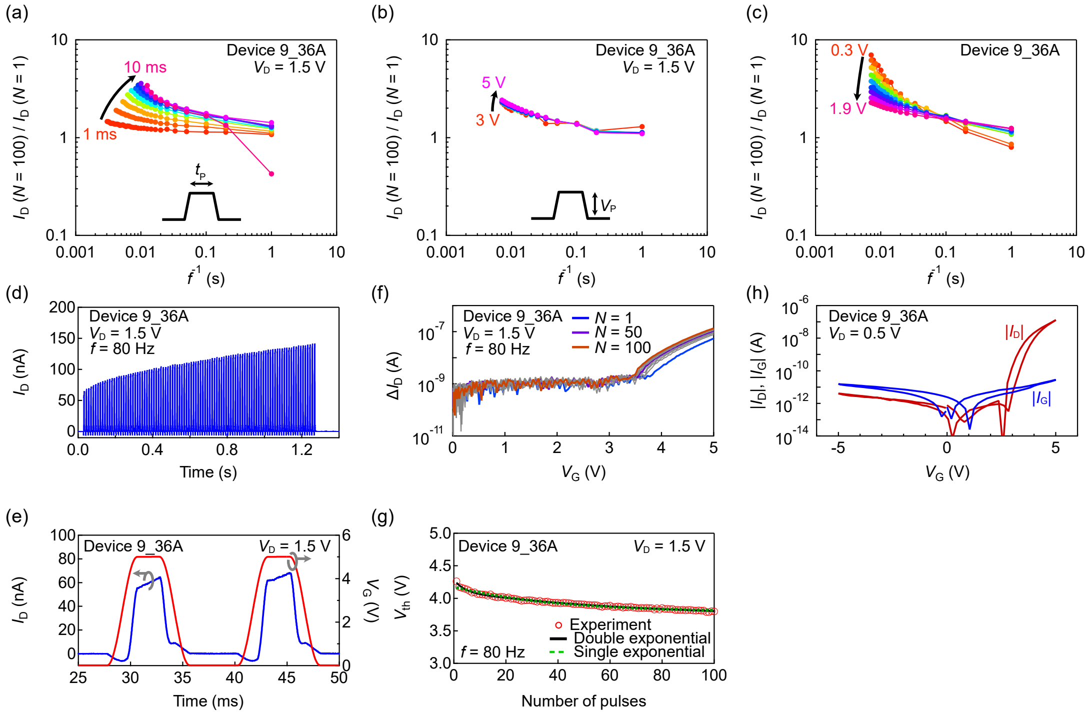

Figure S9: Characterization results of a different Device 9_36A with $L = 9$ µm and $W = 36$ µm. a-c) Frequency dependence of the leaky-integration ratio $I_D(N = 100)/I_D(N = 1)$ for (a) various pulse widths t_p, (b) pulse height V_p, and (c) drain voltages V_D. d) Temporal response of Device 9_36A to the gate voltage pulses. e) Magnified view of (d). f) Pulse-by-pulse $\Delta I_D - V_G$ curves during leaky integration for the different numbers of the pulses (N). ΔI_D is defined as I_D with the displacement current contribution $-C\frac{dV_G}{dt}$ subtracted. g) Shift of the threshold voltage V_{th} extracted from (f) during leaky integration. h) Quasi-static $I_D - V_G$ and $I_G - V_G$ curves of Device 9_36A.

8 X-ray diffraction characterization of the leaky-integration $SrTiO_3$ FET

To gain further information on the material used in the $SrTiO_3$ FET presented in the main text, we characterized the device using the x-ray diffraction (XRD) technique. The XRD pattern of the device presented in the main text is shown in Figure S10. We observed peaks originating from the $SrTiO_3$ substrate, as well as Au, used as electrodes of the device and contacts of the chip carrier on which the sample was mounted. $SrTiO_3$ is oriented in the *c*-axis direction, as exemplified by the observation of $(00L)$ peaks, but we observe small peaks originating from misaligned $SrTiO_3$, possibly caused by device handling processes such as ultrasonic wire bonding. We did not observe peaks originating from Parylene C, HfO_x, and SiO_x used as part of the device structure due to their non-crystalline nature.

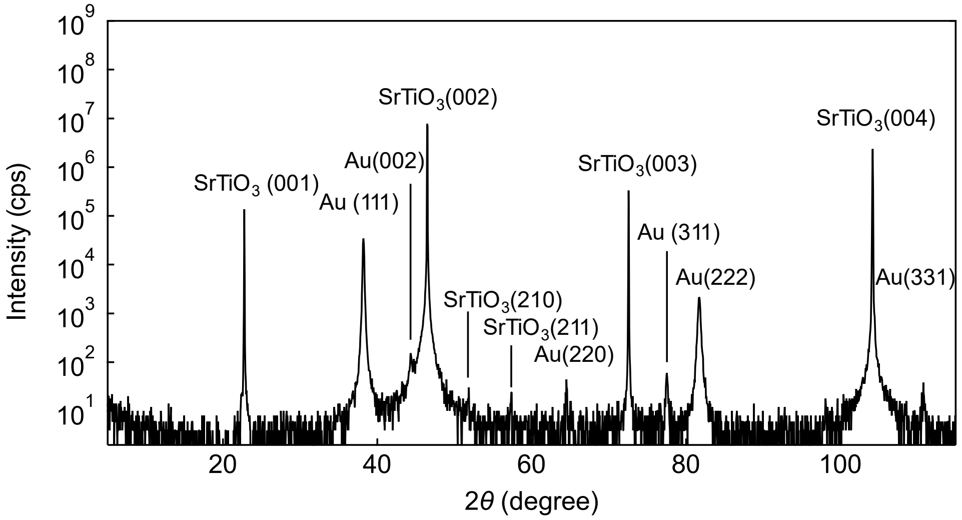

Figure S10: XRD pattern of the device presented in the main text.

9 Energy efficiency and scalability of the leaky-integrating $SrTiO_3$ FET

Compared to devices with a two-terminal (2T) structure, our three-terminal (3T) leaky-integrating $SrTiO_3$ FET has a functional advantage. In the 2T structure, the electrode to control the device's conductivity is identical to the electrode to probe the conductivity. Therefore, applying a control voltage to modify its conductivity also induces the currents dependent on the conductivity of the 2T device. Sometimes, it is necessary to use a large voltage to control the device's conductivity state, resulting in significant energy dissipation by Joule heating. Furthermore, the spurious signal induced by the application of control voltage is sent to the following neuronal devices, causing harm to information processing.

The $SrTiO_3$ FET has a gate electrode to control leaky integration separated from the drain electrode that is used to probe the resulting change in the drain current. No drain voltage is necessary to control the leaky integration of the $SrTiO_3$ FET. Therefore, only a tiny current is required for leaky integration owing to the very high gate resistance. Considering the gate resistance > 30 GOhm of our device, the energy consumed by leaky integration is estimated to be 1.5 pJ/pulse when operated with $V_p = 3$ V and $t_p = 5$ ms. If we assume an average firing rate of 10 Hz in the neural network corresponding to the timescale of 100 ms, the power is estimated to be 15 pW per neuron. This power is significantly smaller than the estimated power of 6 μW of a CMOS neuron [1]. Additionally, neuronal devices connected to the 3T device are free from the spurious signals generated by applications of the control voltage.

Although, in principle, the development of a 2T device with an operation mechanism similar to the leaky-integrating $SrTiO_3$ FET may be possible, the transistor device configuration helps the effective implementation of the leaky-integration function because only a small change in oxygen vacancy concentration is necessary to induce the leaky-integration behavior.

To assess the scalability of the leaky-integrating $SrTiO_3$ FET, we have prepared and characterized devices with different lengths L and widths W of the transistor channel. The characterization results are shown in Figure S6, S7, S8, and S9. In Figure S11a, the evolution of threshold voltage V_{th} of the transistor during the sequence of 80 Hz gate voltage pulse is compared. As described in the main text, V_{th} gradually shifts to smaller voltages as a result of leaky integration. The time scale at which the threshold shifts is related to the leaky-integration time constant of the device. We did not see significant variation in the timescales among these devices. Therefore, the leaky-integration time constant is expected to be independent of the length and width of the transistor channel. The insensitivity of the time constants on device size comes from the fact that the time constant is dominated by the drift-diffusion dynamics of oxygen vacancies and is advantageous to realize long-time constants in smaller devices.

For the device size dependence of the actual value of the V_{th} shown in Figure S11b, an increase in channel lengths and widths tends to increase V_{th}. Therefore, by scaling down the device size, we expect V_{th} can be further decreased, helping to operate the device in smaller gate voltages and contributing to reducing energy consumption.

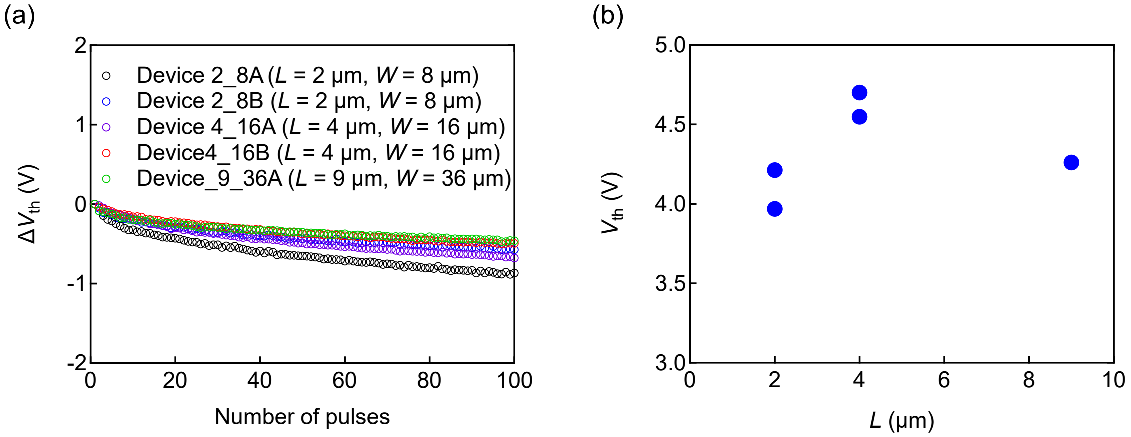

Figure S11: a) Variation of threshold voltage ΔV_{th} as a function of the number of gate pulses during the application of an 80 Hz pulse train. ΔV_{th} is defined as V_{th} with the initial V_{th} subtracted. Device 2_8A is the device presented in the main text. b) Variation of V_{th} as a function of the channel length L.

10 Effect of neuron's timescales on the performance of information processing

In Section 2.1 of the main text, we showed that handwriting anomaly detection is unsuccessful when the neuron's timescale is much shorter than the timescales of handwriting trajectory. For neural network processing tasks that refer to the history of signals, the performance of information processing is generally dependent on the timescales of the constituting neurons when the signals are processed consecutively without external memories.

The underlying reason lies in the neuron's leaky-integration behavior (see Figure S12) – the neuron's membrane potential decays after receiving an input pulse. If the neuron's timescale is longer than the time interval of the pulses ($\tau_n > \tau_{in}$), a finite potential remains when the next pulse arrives. In other words, the neuron remembers that it received an input before. However, if the neuron's timescale is shorter ($\tau_n < \tau_{in}$), the membrane potential completely relaxes before it receives the next pulse. Therefore, the neuron loses the information after time comparable to its leaky-integration timescale is passed.

Many tasks, including handwriting anomaly detection, involve evaluating the time-correlation of input signals, and the information of previous signals must remain within the neural network. The inability to process signals having timescales much longer than the neurons' process ($\tau_n < \tau_{in}$) is indeed exemplified in our demonstration of the handwriting anomaly detection in the main text Figure 2.

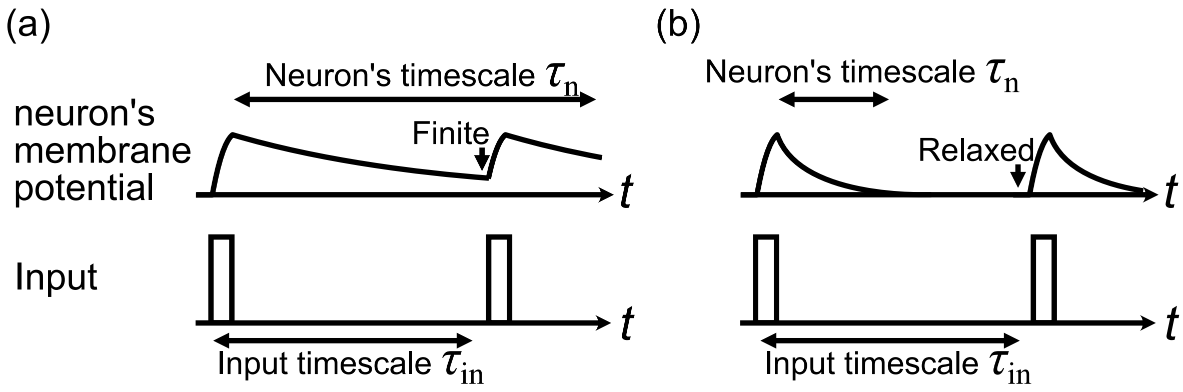

Figure S12: Time evolution of neuron's membrane potential a) when the neuron's timescale τ_n is longer than the input timescale τ_{in}, and b) when τ_n is shorter than τ_{in}

11 Readout function of the reservoir for handwriting anomaly detection

In reservoir-based information processing, the readout layer that encodes the reservoir states to the desired outputs is usually utilized. For the task of handwriting anomaly detection described in the main text Section 2.1, we have employed the readout function that generates the anomaly score S_A (green curves in the main text Figure 2c and d). Since S_A cannot be generated by a simple synapse layer or other layers consisting of neural networks, we calculated S_A numerically from the raw output values of the neural elements (Figure S13a). S_A is given by $S_\mathrm{A} = \boldsymbol{r}(t)^\top P\boldsymbol{r}(t)$ with the precision matrix $P = (\Sigma_0 + \alpha I)^{-1}$ described in the Supplementary Section 4. With 256 neural elements in the reservoir, the calculation of S_A involves matrix multiplication between 256×256 elements and the 256-element vector. This number of matrix multiplication is significantly smaller than the equivalent algorithms such as deep learning, and the method to utilize S_A is robust and advantageous for its anomaly detection performance. Applications requiring a smaller number of calculations than the S_A approach can use the mean-squared-error (MSE) based readout layer implemented below (Figure S13b and S14a).

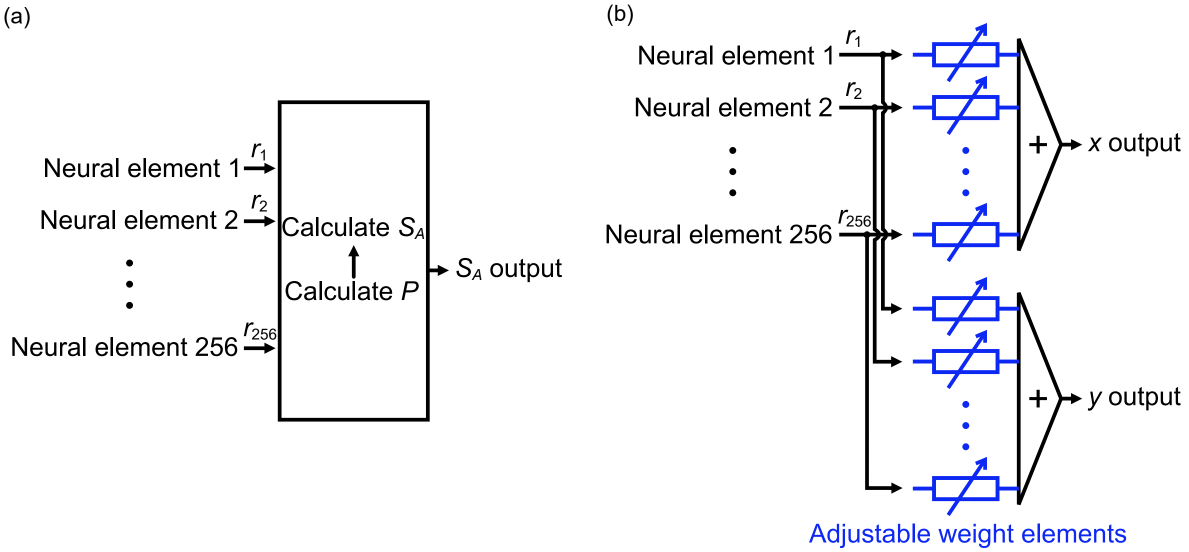

Figure S13: Two different schemes used as the readout function. a) S_A-based readout function, which directly calculates anomaly score S_A from the outputs of the neural elements. b) Adjustable-weight-based readout function, which adjusts weight elements to generate x, y outputs that reproduce the x, y inputs.

The MSE-based readout function is implemented by a single layer of adjustable weight elements, each connected between the synapse in the reservoir and an adder provided for each output (x and y outputs, see Figure S13b). The weight element has a single input and outputs the input value scaled by a programmed amount. Each adder has 256 inputs, which are added together to generate a single output. During the training phase, the weights are trained to reproduce the input x and y signals (Figure S14b). The anomaly is detected by calculating the

$MSE = (x_{in} - x_{out})^2 + (y_{in} - y_{out})^2$. The result is shown in Figure S14c and S14d. For the case of the reservoir with long-timescale neurons ($\tau_n = 1.07$ s), the MSE goes above the threshold only when the different person B's handwriting trajectories are provided to the reservoir. In contrast, for the case of the reservoir with short-timescale neurons ($\tau_n = 10$ μs), the MSE is always below the threshold. Therefore, handwriting anomaly detection is successful only when the long-timescale neurons are used. The results are consistent with when S_A is used as the anomaly score.

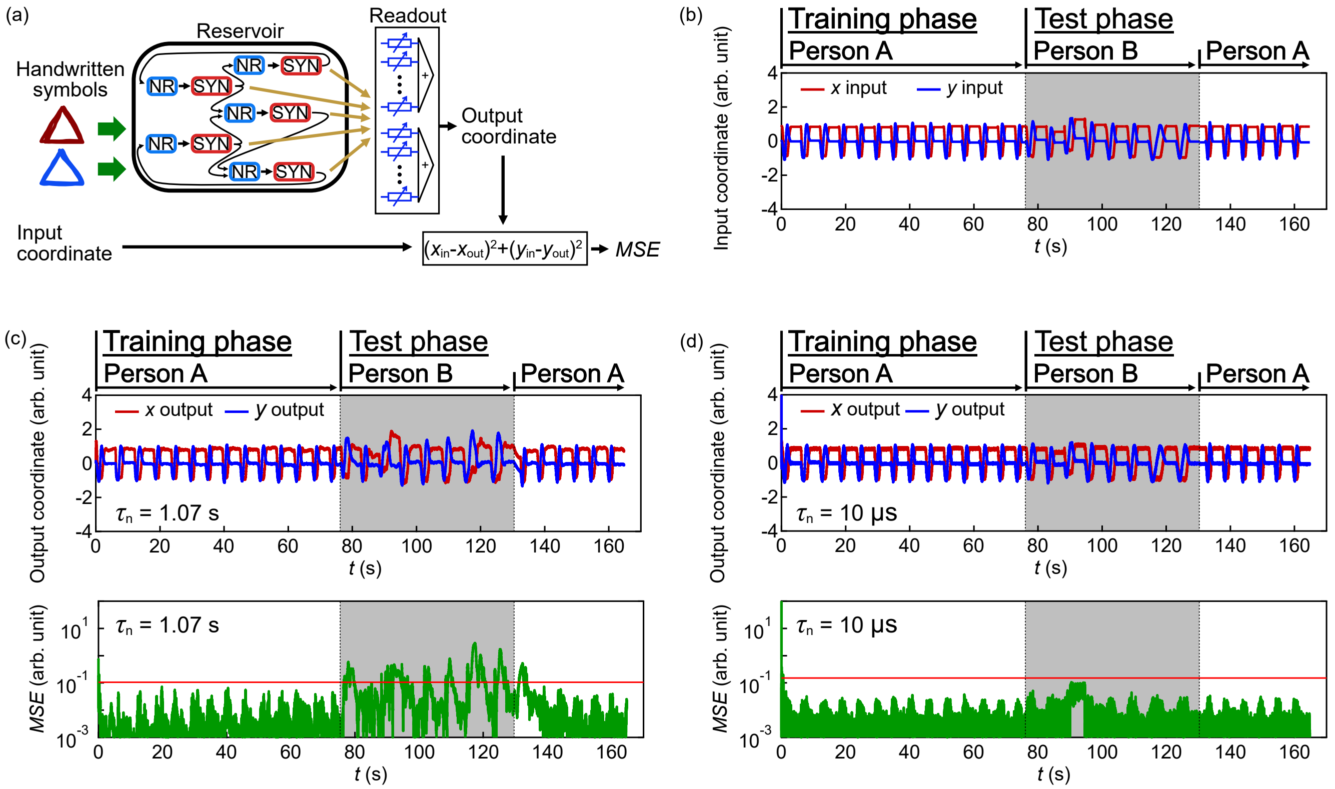

Figure S14: Result of the handwriting anomaly detection using the mean-squared error (MSE) as the anomaly score. a) Configuration of the MSE-based handwriting anomaly detection. b) Input signals corresponding to the trajectory of a pen. c-d) Output signals from the readout function and the corresponding MSE when (c) $\tau_n = 1.07$ s, and (d) $\tau_n = 10$ μs. The red lines in (c) and (d) are the threshold level of the anomaly detection.

12 Modeling the electric field in $SrTiO_3$ during the oxygen vacancy drift-diffusion

It might be counterintuitive that the oxygen vacancies drift towards the substrate direction when the voltage is applied between the laterally placed source/drain and gate electrodes. To help understand why oxygen vacancies migrate toward the substrate direction, we have calculated below the electric field distribution during the gate voltage application (Figure S15, corresponding to $t = 1.0$ ms in the main text Figure 4e II and 4f II). The direction of the electric field $\boldsymbol{E}$ around the center line of the transistor channel is pointing toward the substrate direction. Furthermore, the electric field initially drops by an order of magnitude within 0.3 μm from the surface toward the substrate direction, and it further extends over several μm. The downward direction of the electric field is because of this long extension of the electric fields and the large dielectric constant $300\varepsilon_0$ of $SrTO_3$. As a result, the oxygen vacancies can migrate by the electromagnetic force over several μm toward the substrate direction.

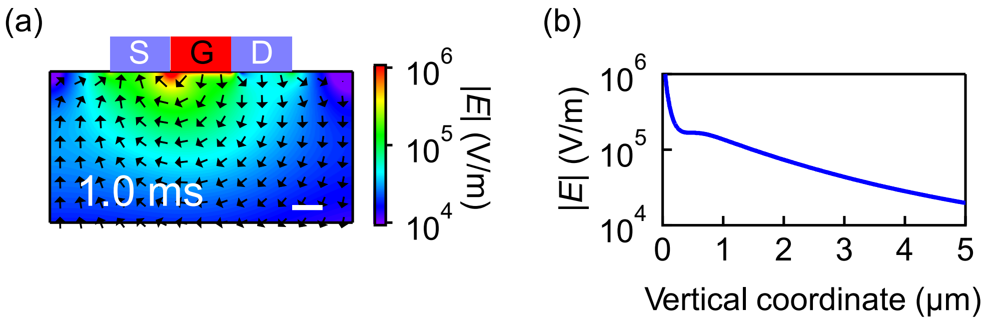

Figure S15: a) Electric field distribution at $t = 1.0$ ms (V_G ramping scheme is the same as in the main text Figure 4d). The color map and the arrows indicate the magnitude and direction of the electric field, respectively. The scale bar is 1 μm. b) Magnitude of the electric field along the center line of the device. The vertical coordinate of 0 μm corresponds to the $SrTiO_3$/Parylene C interface.

13 Endurance of the leaky-integrating $SrTiO_3$ FET

In Figure S16, we show evaluation results of the endurance of the leaky-integrating $SrTiO_3$ FET presented in the main text by applying up to 1.715×10^8 gate pulses corresponding to 95 hours. Figure S16a and S16b show the time evolution of applied gate voltages V_G (top panels), which consist of alternating positive and negative voltage pulses, and the measured drain currents I_D (bottom panels) for different time periods of the endurance test. The I_D during the positive gate pulse is plotted in Figure S16c as a function of the number of applied gate pulses. It initially increases to 130 nA and asymptotically decreases to 30 nA. The increase and decrease of I_D are caused by the effect of positive and negative gate pulses, which move back and forth the oxygen vacancies in the $SrTiO_3$ crystal; leaky integration of the positive applied voltages is counter-balanced by the leaky integration of the negative applied voltages. We did not see a noticeable degradation of the device's performance from $I_D - V_G$ and $I_G - V_G$ curves before and after the endurance test shown in Figure S16d and S16e, respectively. I_G remains at a similar level, and I_D is increased after the test due to the change in the operation points.

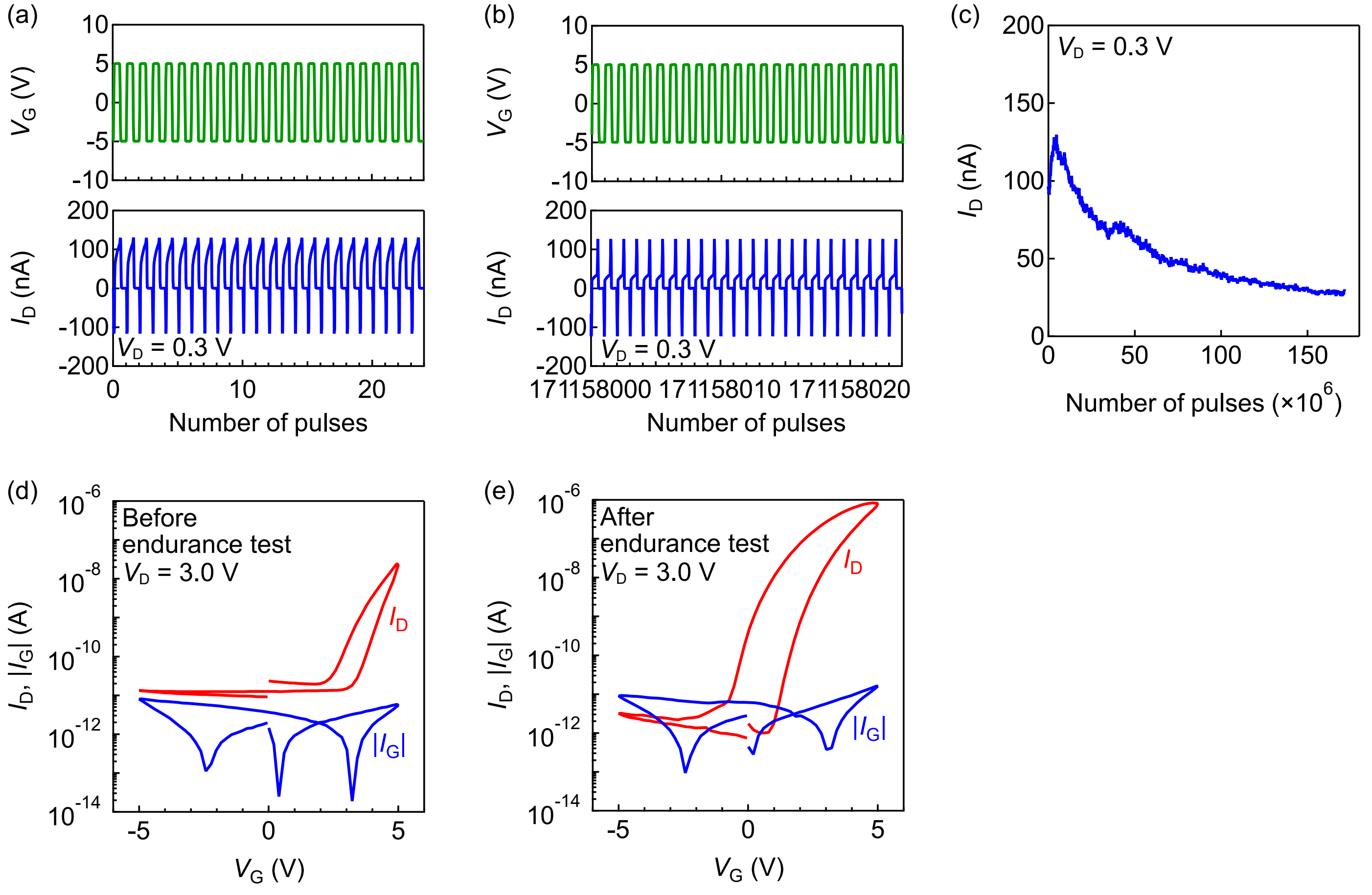

Figure S16: a-b) Time evolution of applied gate voltage V_G and the measured drain current I_D (a) around the initial stage of the test and (b) toward the end of the test. c) I_D during the positive gate pulses as a function of the number of applied gate pulses. d) $I_D - V_G$ and $I_G - V_G$ curves before the endurance test. e) $I_D - V_G$ and $I_G - V_G$ curves after the endurance test.

14 Comparison of emerging leaky-integration devices

In Table S17, we compare our leaky-integration $SrTiO_3$ FET with other experimentally demonstrated capacitor-less emerging leaky-integration devices reported in the literature.

Mechanism	Material	Type*1	Timescale	Pulse width*2	Pulse height*2	Pulse period*2	Typical current w/o V_D*2	Energy consumption w/o V_D*3	Power consumption w/o V_D*4	V_D*2	Typical current w/ V_D*2	Energy consumption w/ V_D*5	Power consumption w/ V_D*6	Area (channel $L \times W$)	Endurance	Reference
Ferroelectric FET	$TaN/Hf_{0.5}Zr_{0.5}O_2/TaN$	3T	0.4 s	5 ms	1.6 V	40 ms	-	-	-	1.8 V		-	-	-	-	[7]
Anti-ferroelectric FET	$TiN/Hf_xZr_{1-x}O_2/TiN$	3T	0.8 ms	1 μs	1.7 V	2 μs	-	-	-	0.3 V	50 nA	37 fJ/spike*7	1.85 nW	10 μm^2	>10^{12}	[8]
Floating gate FET	Graphene/hBN/MoS_2	2T or 3T	1.3 s	100 ms	4 V	4 s	1 nA	400 pJ/spike	100 pW	-	-	-	-	400 μm^2	>10^4	[9]
Protonic resistance change	$Pt/WO_3/Pd$	2T	49 s	5 s	0.45 V (0.5 V at idle state and 0.05 V at pulse)	50 s	20 μA	10 μJ/spike*9	200 nW*8	-	-	-	-	0.03 mm^2	-	[10]
Atomic diffusion and redox	$Pt/Ag/Ag:SiO_2/Ag/Pt$	2T	300 μs ~ 30 ms	50 μs	1 V	100 μs	20 μA	1 nJ/spike	10 μW	-	-	-	-	25 μm^2	>10^6	[11][12]
Mott transition	$GaTa_4Se_8$ (T = 74 K)	2T	518 μs	20 μs	40 V	100 μs	400 μA	320 nJ/spike	3.2 mW	-	-	-	-	-	-	[13]
Phase change	$TiO_2/VO_2/Au$	2T	43 μs	10 μs	10 V	20 μs	1 mA	100 nJ/spike	5 mW	-	-	-	-	-	-	[14]
Ion migration	$Ti/TiO_x/Pt$	2T	40 ms	10 μs	4 V	100 μs	500 nA	20 pJ/spike	200 nW	-	-	-	-	25 μm^2	>5×10^6	[15]
Ion migration	$SiO_2/WO_x/Pd/Au$	2T	50 ms	500 μs	1.4 V	5 ms	1 μA	700 pJ/spike	140 nW	-	-	-	-	0.25 μm^2	-	[16]
Electrolysis	$SrTiO_3/12CaO\cdot7Al_2O_3$	3T	21 s	65 s	10 V	240 s	200 pA	130 nJ/spike	542 pW	2 V	100 μA	13 mJ/spike	54 μW	0.16 mm^2	-	[17]
Ion migration	$ITO/CH_3NH_3PbI_3/Au$	2T	25 ms	5 ms	3 V	25 ms	30 μA	450 nJ/spike	18 μW	-	-	-	-	0.01 mm^2	>4000	[18]
Mott transition	$W/TiN/NbO_2/TiN$ with integrated capacitor	2T	1 μs	50 ns	2.05 V	1 μs	1.25 mA	130 pJ/spike	130 μW	-	-	-	-	0.003 μm^2	-	[19]
Drift diffusion	$SrTiO_3$/ParyleneC/HfO_x	3T	1.07 s	2 ms	5 V	0.1 s	30 pA	300 fJ/spike	3 pW	0.5 V	50 nA	50 pJ/spike	500 pW	16 μm^2	>10^8	This work

*1 2T and 3T stand for two-terminal and three-terminal devices, respectively.
*2 Typical value experimentally demonstrated in the refence and used for the energy and power estimation in this table.
*3 Energy consumption w/o V_D = Pulse width × Pulse height × Typical current w/o V_D unless otherwise indicated.
*4 Power consumption w/o V_D = Energy consumption w/o V_D / Pulse period unless otherwise indicated.
*5 Energy consumption w/ V_D = Pulse width × V_D × Typical current w/ V_D unless otherwise indicated.
*6 Power consumption w/ V_D = Energy consumption w/ V_D / Pulse period unless otherwise indicated.
*7 Value reported in the reference.
*8 Value reported in the reference.
*9 Energy consumption w/o V_D = Power consumption w/o V_D × Pulse period.

Figure S17: Comparison of various parameters among experimentally demonstrated capacitor-less emerging leaky-integration devices reported in the literature [7, 8, 9, 10, 11, 12, 13, 14, 15, 16, 17, 18, 19].

References

[1] X. Chen, T. Yajima, I. H. Inoue, T. Iizuka, *Jpn. J. Appl. Phys.* **2022**, *61*, SC1051.

[2] H. Tamura, K. Fujiwara, K. Aihara, G. Tanaka, *Mahalanobis Distance of Reservoir States for Online Time-Series Anomaly Detection*, (Preprint) techrxiv:22678774, **2023**.

[3] S. A. Chambers, Y. Liang, Z. Yu, R. Droopad, J. Ramdani, K. Eisenbeiser, *Appl. Phys. Lett.* **2000**, *77*, 1662.

[4] Y. Yamada, Y. Kanemitsu, *Phys. Rev. B* **2010**, *82*, 121103.

[5] V. V. Afanas'ev, *Adv. Condens. Matter Phys.* **2014**, *2014*, 301302.

[6] V. Podzorov, M. E. Gershenson, *Phys. Rev. Lett.* **2005**, *95*, 016602.

[7] J. Luo, L. Yu, T. Liu, M. Yang, Z. Fu, Z. Liang, L. Chen, C. Chen, S. Liu, S. Wu, Q. Huang, R. Huang, in *2019 IEEE International Electron Devices Meeting (IEDM)*, IEEE, **2019**, pp. 6–4.

[8] R. Cao, X. Zhang, S. Liu, J. Lu, Y. Wang, H. Jiang, Y. Yang, Y. Sun, W. Wei, J. Wang, H. Xu, Q. Li, Q. Liu, *Nat. Commun.* **2022**, *13*, 7018.

[9] U. Y. Won, Q. An Vu, S. B. Park, M. H. Park, V. Dam Do, H. J. Park, H. Yang, Y. H. Lee, W. J. Yu, *Nat. Commun.* **2023**, *14*, 3070.

[10] S. P. Pati, Y. Geng, S. Hamasuna, K. Fujiwara, T. Iizuka, H. Inoue, I. Inoue, T. Yajima, *Commun. Mater.* **2024**, *5*, 177.

[11] Z. Wang, S. Joshi, S. Savel'ev, W. Song, R. Midya, Y. Li, M. Rao, P. Yan, S. Asapu, Y. Zhuo, H. Jiang, P. Lin, C. Li, J. H. Yoon, N. K. Upadhyay, J. Zhang, M. Hu, J. P. Strachan, M. Barnell, Q. Wu, H. Wu, R. S. Williams, Q. Xia, J. J. Yang, *Nat. Electron.* **2018**, *1*, 137.

[12] Z. Wang, S. Joshi, S. E. Savel'ev, H. Jiang, R. Midya, P. Lin, M. Hu, N. Ge, J. P. Strachan, Z. Li, Q. Wu, M. Barnel, G.-L. Li, H. L. Xin, R. S. Williams, Q. Xia, J. J. Yang, *Nat. Mater.* **2017**, *16*, 101.

[13] P. Stoliar, J. Tranchant, B. Corraze, E. Janod, M.-P. Besland, F. Tesler, M. Rozenberg, L. Cario, *Adv. Funct. Mater.* **2017**, *27*, 1604740.

[14] T. Yajima, T. Nishimura, A. Toriumi, in *2018 IEEE Symposium on VLSI Technology*, **2018**, pp. 27–28.

[15] S.-O. Park, H. Jeong, J. Park, J. Bae, S. Choi, *Nat. Commun.* **2022**, *13*, 2888.

[16] C. Du, F. Cai, M. A. Zidan, W. Ma, S. H. Lee, W. D. Lu, *Nat. Commun.* **2017**, *8*, 2204.

[17] H. Ohta, Y. Sato, T. Kato, S. Kim, K. Nomura, Y. Ikuhara, H. Hosono, *Nat. Commun.* **2010**, *1*, 118.

[18] J.-Q. Yang, R. Wang, Z.-P. Wang, Q.-Y. Ma, J.-Y. Mao, Y. Ren, X. Yang, Y. Zhou, S.-T. Han, *Nano Energy* **2020**, *74*, 104828.

[19] S. Kumar, R. S. Williams, Z. Wang, *Nature* **2020**, *585*, 518.
